# Comparative transcriptomics of primary cells in vertebrates

**DOI:** 10.1101/gr.255679.119

**Published:** 2020-07

**Authors:** Tanvir Alam, Saumya Agrawal, Jessica Severin, Robert S. Young, Robin Andersson, Erik Arner, Akira Hasegawa, Marina Lizio, Jordan A. Ramilowski, Imad Abugessaisa, Yuri Ishizu, Shohei Noma, Hiroshi Tarui, Martin S. Taylor, Timo Lassmann, Masayoshi Itoh, Takeya Kasukawa, Hideya Kawaji, Luigi Marchionni, Guojun Sheng, Alistair R.R. Forrest, Levon M. Khachigian, Yoshihide Hayashizaki, Piero Carninci, Michiel J.L. de Hoon

**Affiliations:** 1 College of Science and Engineering, Hamad Bin Khalifa University, Doha, Qatar;; 2 RIKEN Center for Integrative Medical Sciences, Yokohama 230-0045, Japan;; 3 Centre for Global Health Research, Usher Institute, University of Edinburgh, Edinburgh EH8 9AG, United Kingdom;; 4 MRC Human Genetics Unit, MRC Institute of Genetics and Molecular Medicine, University of Edinburgh, Edinburgh EH4 2XU, United Kingdom;; 5 The Bioinformatics Centre, Department of Biology, University of Copenhagen, 2200 Copenhagen, Denmark;; 6 RIKEN Center for Life Science Technologies, Division of Genomic Technologies, Yokohama 230-0045, Japan;; 7 Telethon Kids Institute, University of Western Australia, Perth, WA 6009, Australia;; 8 RIKEN Preventive Medicine and Diagnosis Innovation Program, Wako 351-0198, Japan;; 9 Department of Oncology, Johns Hopkins University School of Medicine, Baltimore, Maryland 21287, USA;; 10 International Research Center for Medical Sciences (IRCMS), Kumamoto University, Kumamoto 860-0811, Japan;; 11 Harry Perkins Institute of Medical Research, and the Centre for Medical Research, University of Western Australia, QEII Medical Centre, Perth, WA 6009, Australia;; 12 Vascular Biology and Translational Research, School of Medical Sciences, Faculty of Medicine, University of New South Wales, Sydney, NSW 2052 Australia

## Abstract

Gene expression profiles in homologous tissues have been observed to be different between species, which may be due to differences between species in the gene expression program in each cell type, but may also reflect differences in cell type composition of each tissue in different species. Here, we compare expression profiles in matching primary cells in human, mouse, rat, dog, and chicken using Cap Analysis Gene Expression (CAGE) and short RNA (sRNA) sequencing data from FANTOM5. While we find that expression profiles of orthologous genes in different species are highly correlated across cell types, in each cell type many genes were differentially expressed between species. Expression of genes with products involved in transcription, RNA processing, and transcriptional regulation was more likely to be conserved, while expression of genes encoding proteins involved in intercellular communication was more likely to have diverged during evolution. Conservation of expression correlated positively with the evolutionary age of genes, suggesting that divergence in expression levels of genes critical for cell function was restricted during evolution. Motif activity analysis showed that both promoters and enhancers are activated by the same transcription factors in different species. An analysis of expression levels of mature miRNAs and of primary miRNAs identified by CAGE revealed that evolutionary old miRNAs are more likely to have conserved expression patterns than young miRNAs. We conclude that key aspects of the regulatory network are conserved, while differential expression of genes involved in cell-to-cell communication may contribute greatly to phenotypic differences between species.

Vertebrate organisms consist of hundreds of cell types, with more than 400 cell types defined in human (Vickaryous and Hall 2006). Traditionally, cell types have been defined by their tissue of origin as well as by their cellular phenotypes including morphology, staining properties, enzyme histochemistry, and cell surface marker recognition by antibodies (Vickaryous and Hall 2006). Cell type characterization has been supplemented by molecular approaches such as molecular fingerprinting (Arendt 2008) as well as genome-wide profiling of the transcriptome of primary cells (The FANTOM Consortium and the RIKEN PMI and CLST (DGT) 2014). To this end, the Human Cell Atlas initiative aims to comprehensively define human cell types by performing transcriptome analysis in single cells on a massive scale (Regev et al. 2017).

Evolution of anatomy is thought to primarily depend on the evolution of gene expression patterns and regulation, rather than the evolution of the encoded protein sequences (Britten and Davidson 1971; King and Wilson 1975). While comparative studies have shown that gene expression programs in matching tissues are largely conserved between species (Su et al. 2002; Chan et al. 2009; Brawand et al. 2011; Merkin et al. 2012), many genes were found to be differentially expressed (Su et al. 2002; Lin et al. 2014; Yue et al. 2014). Although such expression differences between human and mouse for specific genes may be due in part to differences in cell type composition of the analyzed tissues (Breschi et al. 2017), little overlap was found in terms of differentially expressed genes between human and mouse in dynamic studies of primary cells during erythropoiesis (Pishesha et al. 2014) and of primary macrophages upon stimulation by lipopolysaccharide (Schroder et al. 2012) or by glucocorticoid (Jubb et al. 2016). Collectively, these findings suggest that also in matching primary cells many genes are differentially expressed between species. As cells with an identical cellular phenotype may display distinct and disparate molecular phenotypes, the question of what key transcriptomic features define a cell type is raised (Arendt et al. 2016).

The confounding effects of cell type composition in tissue-based studies can be avoided by comparing the transcriptome of different species in homologous primary cells. Here, we present a comparative analysis of genome-wide expression in vertebrate species profiled in FANTOM5 (The FANTOM Consortium and the RIKEN PMI and CLST (DGT) 2014; Lizio et al. 2017a,b) to elucidate patterns of gene expression conservation during evolution.

## Results

**Figure 1.**
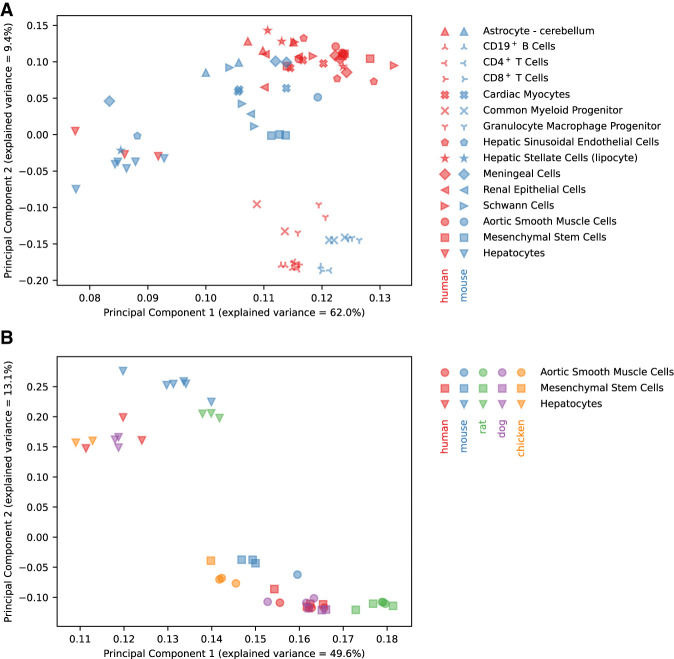
Gene expression PCA. (*A*) PCA for all samples of cell types in common between human and mouse. (*B*) PCA for all samples of cell types in common between all five species.

The FANTOM5 collection contains Cap Analysis Gene Expression (CAGE) data for three primary cell types in human, mouse, rat, dog, and chicken, and for an additional 12 cell types in human and mouse only (Supplemental Table S1). We identified 15,538, 14,915, 13,759, and 8696 protein-coding genes in mouse, rat, dog, and chicken, respectively, with a one-to-one orthologous gene in human, and 6561 protein-coding genes with one-to-one orthologs in all five species (see Methods for details). Principal Component Analysis (PCA) of all human and mouse samples revealed a liver-specific cluster, a mesenchymal cluster, and a hematopoietic cluster (Fig. 1A), and similarly, PCA for cell types with CAGE data available in all five species showed a hepatocyte cluster and a mesenchymal cluster (Fig. 1B). Within each cluster, samples tended to cluster by species (Fig. 1), consistent with the “species signal” phenomenon observed previously (Musser and Wagner 2015).

**Figure 2.**
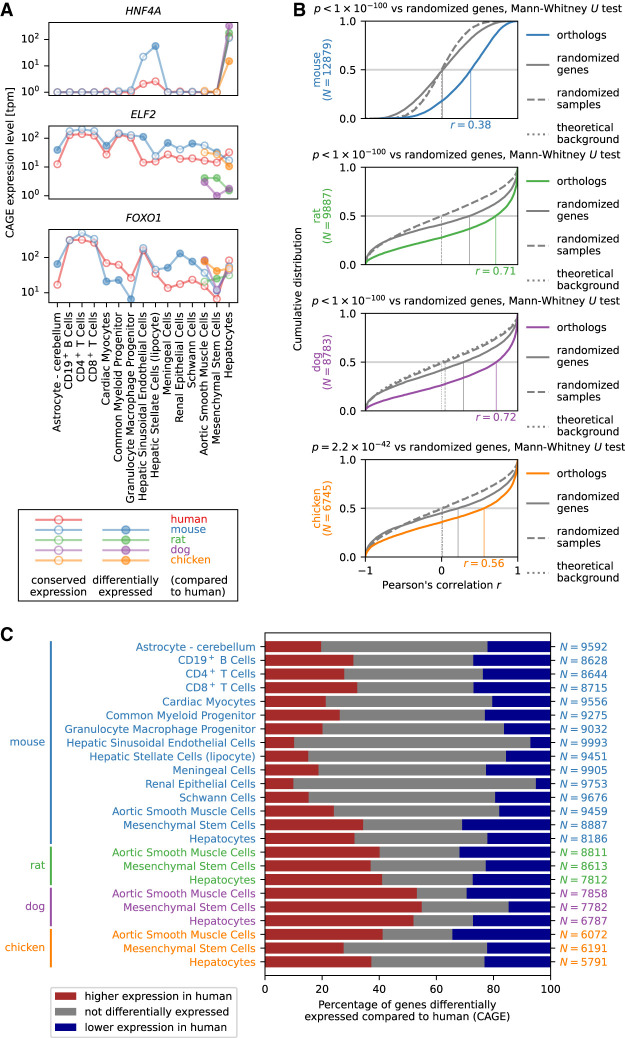
Differential gene expression analysis. (*A*) Expression profile of *HNF4A*, *ELF2*, and *FOXO1* as examples of genes with an expression profile highly correlated across cell types between species but with significant expression level differences between species in specific cell types. (*B*) Cumulative distribution of Pearson's correlation *r* across cell types in gene expression between human and mouse, rat, dog, or chicken. The number *N* of expressed orthologous genes included in the distribution is shown in the vertical axis label, and the estimated median value of *r* is indicated on the horizontal axis of each graph. The background distribution of *r* obtained by randomizing genes (solid curve) or randomizing samples (dashed curve) as well as the theoretical background distribution of *r* for an uncorrelated bivariate normal distribution (dotted curve) are shown in gray; the latter two largely coincide. The statistical significance was calculated using the Mann–Whitney *U* test comparing Pearson's correlation values for orthologs to the background distribution of *r* for randomly paired genes between human and mouse, rat, dog, or chicken. Note that the median correlation values are not directly comparable between species, as the sets of orthologous genes are different. (*C*) Differential gene expression analysis of orthologous genes in human compared to mouse, rat, dog, and chicken. The red and blue bars correspond to the percentage of expressed orthologous genes with significantly (Benjamini–Hochberg corrected *P* < 0.1) higher and lower expression, respectively, in human compared to mouse, rat, dog, or chicken. The number *N* of orthologous genes expressed in each cell type is shown on the *right*.

Expression levels of pairs of orthologous genes were positively correlated across cell types, with median Pearson's correlation values ranging from 0.38 to 0.72 (*P* < 10^−100^, mouse, rat, and dog; *P* = 2.2 × 10^−42^, chicken) (Fig. 2A,B). Nevertheless, in specific cell types we found significant differences in expression of orthologs in different species (Fig. 2A,C). Pairwise differential expression analysis between genes in human and their orthologs in mouse, rat, dog, or chicken for each primary cell type in FANTOM5 revealed that, on average, 52% of expressed genes were differentially expressed (Benjamini–Hochberg corrected *P* < 0.1) between the two species (Fig. 2C; Supplemental Table S2).

**Figure 3.**
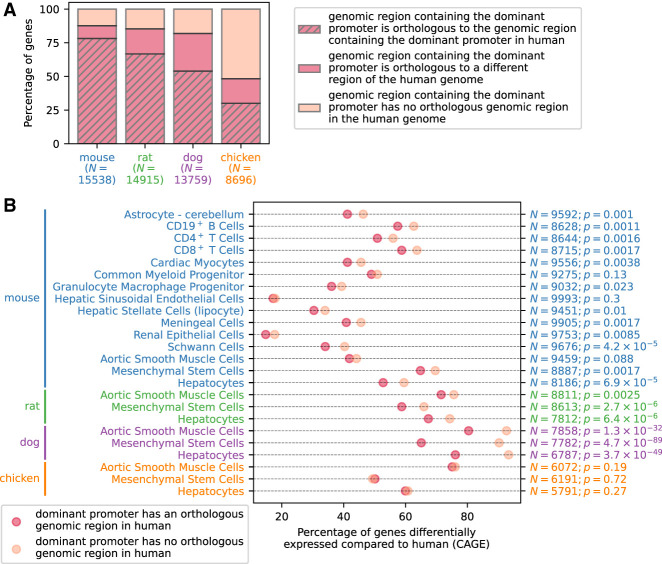
Promoter analysis of differentially expressed genes. (*A*) Percentage of genes in mouse, rat, dog, and chicken for which the dominant promoter was located in a genome region that had an orthologous genome region in human, and the percentage that the orthologous region contained the dominant promoter for the orthologous gene in human. (*B*) Percentage of differentially expressed genes in each cell type depending on whether the genomic region of the dominant promoter in each species had an orthologous genomic region in the human genome. The one-sided *P*-value calculated using Fisher's exact test is shown on the right, together with the number *N* of expressed genes in each cell type.

In each species, we defined the dominant promoter for each gene as the most highly expressed promoter associated with the gene. The genomic region of the dominant promoter of more than 80% of genes in mouse, rat, and dog and 50% of genes in chicken had an orthologous region in the human genome; the majority of those overlapped the corresponding human dominant promoter (Fig. 3A). Genes were more likely to be differentially expressed if their dominant promoter was located in a genomic region that did not have an orthologous genome sequence in the human genome (Fisher combined *P* < 10^−100^) (Fig. 3B; Supplemental Fig. S1), suggesting that gain or loss of promoter sequence regions during evolution contributes to the emergence of gene expression differences between species.

**Figure 4.**
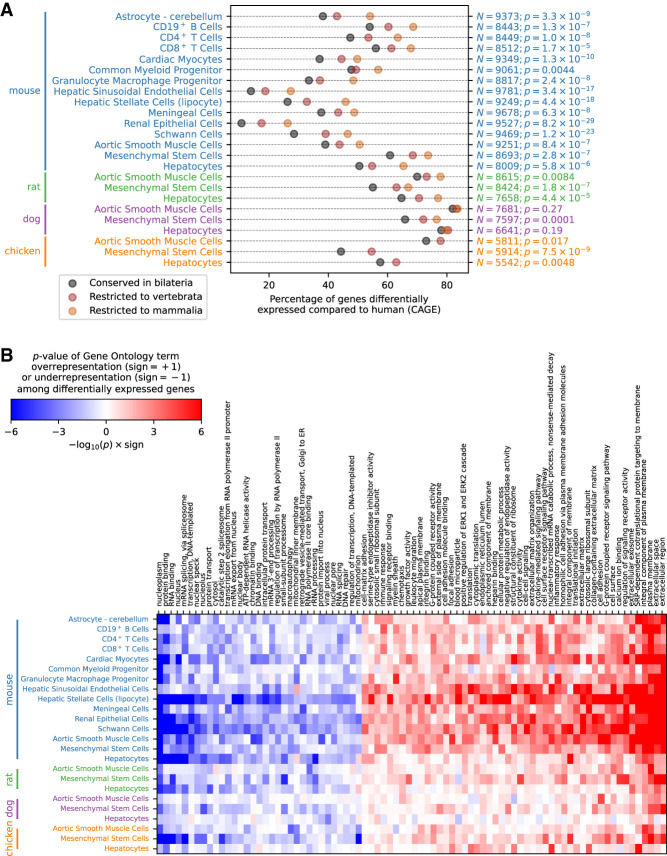
Conservation and Gene Ontology analysis of differentially expressed genes. (*A*) Percentage of differentially expressed genes in each cell type as a function of age of the most recent common ancestor. The one-sided *P*-value of a Poisson regression model against the evolutionary age category is shown on the right, together with the number *N* of expressed genes in each cell type with an annotation in the NCBI HomoloGene database. (*B*) Gene Ontology analysis of differentially expressed genes. The *P*-value, calculated using Fisher's exact test, of overrepresentation or underrepresentation of differentially expressed genes in each Gene Ontology category compared to an expression-matched set of background genes is shown in red and blue, respectively.

We hypothesized that genes critical for cellular functioning would both be more conserved and their expression patterns less diverged during evolution, and indeed we found the expression levels of evolutionarily older genes to be more conserved (Fisher combined *P* < 10^−100^) (Fig. 4A; Supplemental Fig. S2). Gene Ontology analysis of differentially expressed genes showed that genes with products involved in transcription, RNA processing, and transcriptional regulation were more likely to have conserved expression levels, whereas genes encoding proteins localized to the plasma membrane and extracellular space as well as signaling proteins were most likely to be differentially expressed (Fig. 4B; Supplemental Table S3). This suggests that the transcriptional program in each cell tends to be conserved during evolution, while genes in the periphery of the transcriptional regulatory network, especially those involved in cellular communication, tend to diverge in expression.

As an independent confirmation, we applied integrative correlation analysis (Parmigiani et al. 2004) by first calculating the correlations across cell types between all genes for human and mouse separately, and then the correlation across orthologous genes between corresponding rows in these two correlation matrices. This yielded the correlation-of-correlations, or integrative correlation coefficient, as a measure of the degree of expression conservation during evolution for each gene. We then ranked genes based on their integrative correlation coefficient and performed gene set enrichment analysis to identify biological processes most conserved or most divergent between the two species (see Methods section). The integrative correlation coefficient values ranged between −0.52 and 0.59, and their observed distribution was skewed to the right, with a median of 0.25 (Supplemental Fig. S3A; Supplemental Table S4), suggesting that, overall, gene expression profiles tend to be conserved between human and mouse. Similar to our conclusions for Gene Ontology analysis of differentially expressed genes, fundamental cellular processes involved in cell homeostasis and maintenance tended to rank higher in integrative correlation analysis, while gene sets encompassing processes associated with cell-to-cell signaling and other biological processes taking place in the extracellular space (e.g., neuronal and synapse development) were more likely to rank lower, suggesting their underlying networks to be less conserved (Supplemental Fig. S3B; Supplemental Table S4).

As a complement to the differential gene expression analysis, we calculated the expression correlation across genes for each cell type and species. Expression levels were positively correlated within each species as well as between species for related cell types (Supplemental Figs. S4, S5), suggesting that the relative ranking of genes by their expression level tends to be conserved. The correlation value decreased exponentially as a function of phylogenetic distance between species and dropped off most rapidly for mesenchymal stem cells compared to aortic smooth muscle cells and hepatocytes (Supplemental Fig. S6). Consistent with the differential gene expression results, expression levels were more highly correlated for genes for which the dominant promoter had an orthologous genome region in human compared to genes for which the dominant promoter did not have an orthologous genome region (Fisher combined *P* < 10^−100^) (Supplemental Figs. S7, S8), as well as for evolutionarily ancient genes compared to recent genes (Fisher combined *P* < 10^−100^) (Supplemental Figs. S9, S10). A Gene Ontology analysis of correlation values again showed that genes with functional roles associated with RNA biology in the nucleus tended to have conserved expression levels, while genes with functions associated with the plasma membrane, extracellular space, and signaling had lower correlation values (Supplemental Fig. S11; Supplemental Table S3).

To confirm these findings in an independent gene expression data set, we performed differential expression analysis on previously published RNA-seq expression data for endometrial stromal fibroblast primary cells in human, rat, rabbit, ferret, cow, and opossum (Kin et al. 2016). We again found that evolutionarily ancient genes were more likely to have conserved expression levels compared to recent genes (Fisher combined *P* = 1.0 × 10^−11^) (Supplemental Fig. S12A,B). Results of Gene Ontology analysis of differentially expressed genes for these data were highly consistent with those observed in the FANTOM5 samples (Supplemental Fig. S12C), including evidence of rapid evolution of signaling pathways as observed previously (Kin et al. 2016). A comparative analysis of RNA-seq expression data in matching tissues in human and mouse (The ENCODE Project Consortium 2012) also showed preferential conservation of expression levels of evolutionarily ancient genes (Supplemental Fig. S13A,B) and yielded similar patterns of Gene Ontology enrichment (Supplemental Fig. S13C).

**Figure 5.**
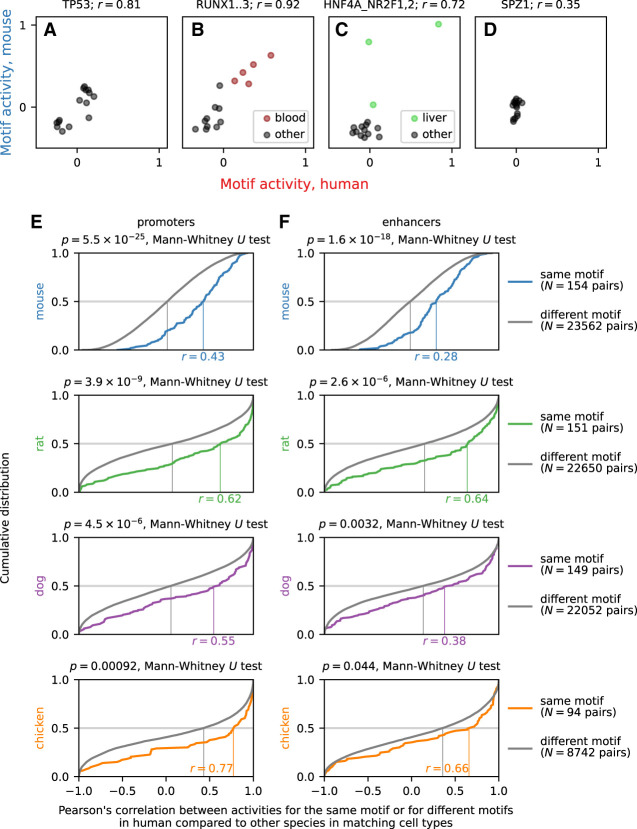
Motif Activity analysis. (*A*–*D*) Examples of calculated motif activities in human and mouse for motifs associated with the broadly expressed transcription factor TP53 (*A*), the hematopoietic lineage-specific RUNX transcription factors (*B*), the hepatocyte-specific HNF4A transcription factor (*C*), and the testis-specific transcription factor SPZ1 (*D*). Each of the 15 matching cell types between human and mouse is shown as a dot. The blood cell types CD19^+^ B cells, CD4^+^ T cells, CD8^+^ T cells, common myeloid progenitors, and granulocyte macrophage progenitors are shown in red for the RUNX motif, and the liver cell types hepatic sinusoidal endothelial cells, hepatic stellate cells (lipocytes), and hepatocytes are shown in green for the motif associated with HNF4A. (*E*,*F*) Cumulative distribution of Pearson's correlation *r* across cell types in motif activity for promoters (*E*) and enhancers (*F*) between human and mouse, rat, dog, and chicken. The estimated median value of *r* is indicated on the horizontal axis of each graph. As a background distribution, we calculated the same correlation between pairs of different motifs in human and mouse, rat, dog, and chicken. The Mann–Whitney *U* test *P*-value comparing the actual correlation values to the correlation values of the background distribution is shown for each comparison.

To understand how evolution of the transcriptional regulatory network affects evolution of gene expression, we used the MotEvo sequence motif analysis software (Arnold et al. 2012) for the 190 motifs compiled in SwissRegulon (Pachkov et al. 2013) to identify potential transcription factor binding sites (TFBSs) in the human, mouse, rat, dog, and chicken genomes. We evaluated the TFBS prediction accuracy using ChIP-seq data (Supplemental Table S5) for transcription factors associated with each motif (Supplemental Fig. S14). Conservation between species of the expression patterns of orthologous genes depended on the concordance in TFBS presence in the promoter of each gene (Supplemental Fig. S15), demonstrating the contribution of *cis*-regulatory evolution to expression divergence between species. To analyze *trans*-regulatory evolution, we performed motif activity analysis (FANTOM Consortium and Riken Omics Science Center 2009), which uses linear decomposition of genome-wide gene expression patterns based on the TFBSs found in the promoter of each gene, resulting in motif activities representing the average expression level of genes with a predicted binding site for each motif. Figure 5 shows the broadly expressed transcription factor TP53 (Fig. 5A), the hematopoietic lineage-specific RUNX transcription factors (Fig. 5B), and the motif associated with the hepatocyte-specific HNF4A transcription factor (Fig. 5C) as examples of motifs with activities highly correlated between human and mouse. In contrast, the motif associated with the testis-specific transcription factor SPZ1 did not show evidence of activation either in human or mouse, as testis was not included in our samples (Fig. 5D). In general, motif activities were highly correlated across samples between human and mouse (*P* = 5.5 × 10^−25^, Mann–Whitney *U*test), rat (*P* = 3.9 × 10^−9^), dog (*P* = 4.5 × 10^−6^), and chicken (*P* = 9.2 × 10^−4^), compared to randomized pairs of motifs (Fig. 5E; Supplemental Table S6).

We then asked if enhancers likewise were activated by the same transcription factors in different species. Enhancers were previously identified in human and mouse from FANTOM5 CAGE data by searching for a characteristic bidirectional expression pattern (Andersson et al. 2014). We predicted enhancers in rat, dog, and chicken by applying the same pipeline on the FANTOM5 CAGE data in these species (Supplemental Table S7) and used the CAGE expression level at each enhancer as a measure of its activity (Andersson et al. 2014). For each species, the motif activity calculated from gene promoter expression profiles correlated with the motif activity based on enhancer expression profiles (human, *P* = 1.2 × 10^−20^, Mann–Whitney *U* test), mouse (*P* = 5.6 × 10^−22^), rat (*P* = 5.6 × 10^−5^), dog (*P* = 2.5 × 10^−5^), and chicken (*P* = 5.7 × 10^−4^) (Supplemental Fig. S16), indicating that, in each species, enhancers are activated by the same transcription factors as promoters. Between species, the motif activity calculated from enhancer expression profiles were correlated between human and mouse (*P* = 1.6 × 10^−18^, Mann–Whitney *U* test), rat (*P* = 2.6 × 10^−6^), dog (*P* = 0.0032), and chicken (*P* = 0.044) (Fig. 5F; Supplemental Table S6). We conclude that both promoters and enhancers are activated by the same transcription factors in different species.

**Figure 6.**
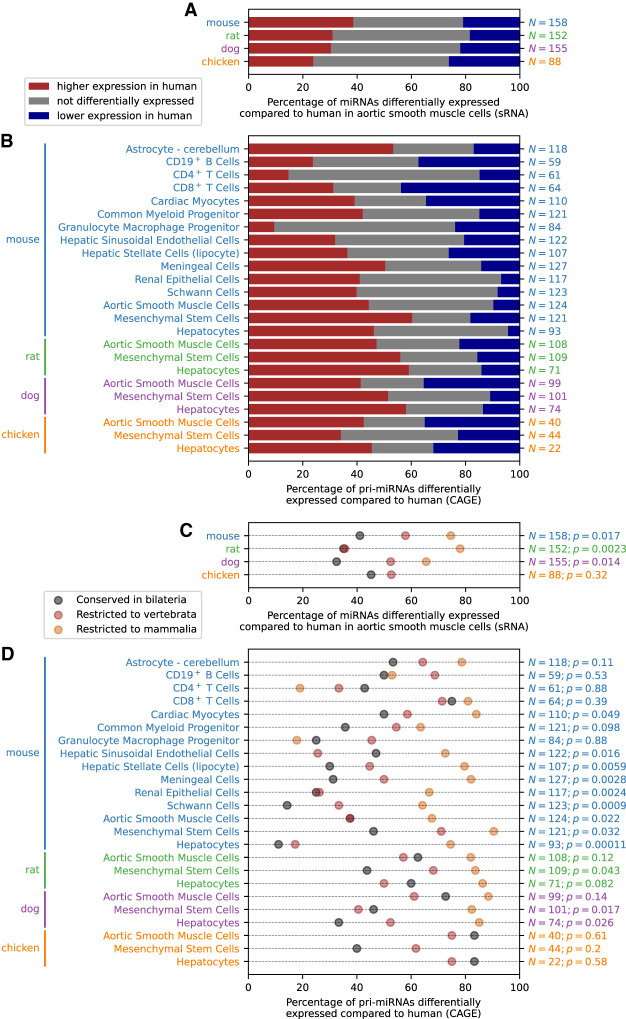
Differential miRNA expression analysis. (*A*) Differential expression analysis of miRNAs using FANTOM5 sRNA sequencing data in aortic smooth muscle cells in human compared to mouse, rat, dog, or chicken. The red and blue bars correspond to the percentage of expressed orthologous miRNAs with significantly (Benjamini–Hochberg corrected *P* < 0.1) higher and lower expression, respectively, in human compared to mouse, rat, dog, or chicken. The number *N* of expressed orthologous miRNAs in each comparison is shown on the right. (*B*) Differential expression analysis of miRNAs in human compared to mouse, rat, dog, and chicken; using CAGE expression of the pri-miRNA as a proxy for the expression level of the mature miRNA. The red and blue bars correspond to the percentage of expressed orthologous miRNAs with significantly (Benjamini–Hochberg corrected *P* < 0.1) higher and lower expression, respectively, in human compared to mouse, rat, dog, or chicken. The number *N* of expressed orthologous miRNAs in each comparison is shown on the *right*. (*C*) Percentage of miRNAs differentially expressed in each comparison, separately based on the evolutionary age of each miRNA. The one-sided *P*-value of a Poisson regression model against the evolutionary age category is shown on the *right*, together with the number *N* of expressed orthologous miRNAs in each comparison. (*D*) Percentage of miRNAs differentially expressed in each comparison, separately based on the evolutionary age of each miRNA; using CAGE expression of the pri-miRNA as a proxy for the expression level of the mature miRNA. The one-sided *P*-value of a Poisson regression model against the evolutionary age category is shown on the right, together with the number *N* of expressed orthologous miRNAs in each comparison.

Next, we extended our comparative analysis to the expression levels of microRNAs (miRNAs). miRNAs are small noncoding RNA (typically 22 nt) that silence mRNA post-transcriptionally and regulate biological processes such as cell growth and differentiation by functional effects on direct targets and regulatory networks (Bracken et al. 2016). In the FANTOM5 collection, short RNA (sRNA) sequencing data for matching primary cell types in different species were available for aortic smooth muscle cells (Supplemental Table S1; Supplemental Table S8). We annotated known (Supplemental Table S9) and candidate novel (Supplemental Table S10) miRNAs in rat, dog, and chicken in the same way as done previously (De Rie et al. 2017) for human and mouse. Differential expression analysis between human and mouse, rat, dog, or chicken showed that about half of the orthologous miRNAs had statistically significant different expression levels in the two species (Fig. 6A; Supplemental Table S11). Dividing miRNAs into three categories based on their evolutionary age revealed that evolutionarily older miRNAs were more likely to have conserved expression levels than younger miRNAs (Fisher combined *P* = 1.2 × 10^−4^) (Fig. 6C).

Previously, we showed that CAGE data can be used to reliably infer the promoter of the primary miRNA (pri-miRNA) transcript and that the corresponding CAGE expression levels can be used as a proxy for the expression level of the mature miRNA (De Rie et al. 2017). We manually curated pri-miRNA promoters previously identified computationally for mouse (De Rie et al. 2017) and, using the same approach, identified pri-miRNA promoters for miRNAs in rat, dog, and chicken (Supplemental Table S12). In aortic smooth muscle cells, expression levels of the mature miRNA measured by sRNA sequencing correlated with the CAGE expression level of the pri-miRNA for mouse, rat, dog, and chicken (Supplemental Fig. S17). The curated primary miRNA promoter annotations as well as expression levels of the mature and primary miRNA are visualized and available for download through an interactive web interface at https://fantom.gsc.riken.jp/zenbu/reports/#FANTOM_miRNA_atlas.

Using these promoters together with previously curated pri-miRNA promoters for human (De Rie et al. 2017), we performed differential expression analysis of miRNAs in human compared to mouse, rat, dog, and chicken. In aortic smooth muscle cells in mouse, rat, dog, and chicken, log-ratios of mature miRNA expression levels, as measured by sRNA sequencing, correlated well with the log-ratios for pri-miRNAs, as measured by CAGE expression data (Supplemental Fig. S18), and among the miRNAs differentially expressed in both data sets, more than 80% showed concordant up- or down-regulation of the mature miRNA and the pri-miRNA, suggesting that few of the identified differentially expressed miRNAs were false positives (Supplemental Fig. S18).

Differential CAGE expression analysis of pri-miRNAs revealed that the majority of expressed orthologous miRNAs have different expression levels in human compared to mouse, rat, dog, and chicken (Fig. 6B; Supplemental Table S13), consistent with the results obtained for mature miRNAs (Fig. 6A). We found significantly fewer differentially expressed miRNAs for evolutionarily old miRNAs compared to evolutionarily recent miRNAs for 12 out of 24 pairwise comparisons, a further seven showed the same pattern without reaching statistical significance, five showed an opposite pattern without reaching statistical significance, and none showed a statistically significant opposite pattern (Fisher combined *P* = 4 × 10^−12^) (Fig. 6D). Therefore, using CAGE as a proxy for miRNA expression allowed us to demonstrate that the patterns observed for mature miRNAs by sRNA sequencing for a single cell type (Fig. 6C) can be found across a wide variety of cell types.

## Discussion

Comparative studies have shown considerable differences in the gene expression levels in matching tissues of different species (Su et al. 2002; Lin et al. 2014; Yue et al. 2014), which is due, at least in part, to differences in tissue composition between species (Breschi et al. 2017). However, our analysis reveals that this cannot be the sole explanation, as considerable expression level differences are also observed between matching primary cell types, indicating that the same cellular phenotype associated with traditionally defined cell types can be achieved by widely different molecular networks.

Our findings suggest that expression levels of regulators tend to be conserved across species, while genes peripheral in the regulatory network, especially those involved in cellular communication, are more likely to have divergent expression patterns. Previously reported examples include the terminal differentiation of erythroid precursors from early to late erythroblasts, where the same transcriptional regulators and other proteins important for erythropoiesis were induced or repressed in human and mouse, suggesting that the core regulatory program of erythroid differentiation remained conserved (Pishesha et al. 2014). In contrast, genes regulated during development showed a different response between human and mouse (Pishesha et al. 2014), indicating that the response of genes to the regulators of erythropoiesis had evolved since the evolutionary split of human and mouse. Similarly, comparing lipopolysaccharide-stimulated macrophages between human and mouse showed enriched differences in the transcriptome of genes encoding proteins involved in cellular communication such as cell surface receptors, inflammatory cytokines, chemokines, and their intracellular signaling pathways (Schroder et al. 2012). Phenotypic differences between species at the organismal level may thus be primarily due to differences in the interaction between cells (Ramilowski et al. 2015).

Orthologous transcription factors typically recognize the same DNA sequence motif in human and mouse (Cheng et al. 2014), as changes in the consensus motif during evolution would simultaneously affect a large number of genes and may be too disruptive. By the same argument, we can expect expression levels of transcriptional regulators to be conserved between species. As a salient example of the conservation of regulatory programs, we previously found that human enhancer sequences could be activated by orthologous transcription factors in corresponding tissues in human and zebrafish (Andersson et al. 2014). In contrast, genomic binding sites of conserved transcription factors have diverged extensively between human and mouse (Odom et al. 2007), suggesting a rewiring of the peripheral regulatory network during evolution.

Due to their modular nature, enhancer regulatory elements are particularly amenable to rewiring, as their cell type- and state-specific usage (Andersson et al. 2014) allows changes in their regulatory connections in specific conditions while avoiding pleiotropic deleterious effects on the organism in general (Carroll 2008). For example, differences in the transcriptome response of human and mouse primary macrophages stimulated by glucocorticoid were previously found to be associated with the turnover of glucocorticoid receptor binding sites at enhancers (Jubb et al. 2016). Similarly, the cell type- and state-specific usage of different promoters associated with a gene (The FANTOM Consortium and the RIKEN PMI and CLST (DGT) 2014) avoids the constraints placed by pleiotropy and allows gain and loss of promoters to contribute significantly to the evolution of species (Young et al. 2015). In our analysis, we indeed find that changes in gene expression levels are associated with the gain and loss of promoter sequence regions during evolution.

Our analysis further shows that the conservation of regulatory programs is not limited to transcriptional regulation but extends to miRNAs. Our comparative analysis of miRNA expression revealed that older miRNAs are more likely to have conserved expression levels than more recent miRNAs, suggesting that highly conserved miRNAs have stronger evolutionary constraints on their expression levels. As an example, we found conservation across human, mouse, rat, dog, and chicken of pri-miRNA expression levels in aortic smooth muscle cells of miR-22, which modulates a range of target genes including *MECP2*, *HDAC4*, and *MECOM* and is a key regulator of smooth muscle cell phenotype switching and neointima formation (Yang et al. 2018).

The Human Cell Atlas aims to create a comprehensive map of cell types in the human body by profiling gene expression levels in single cells from healthy tissues (Regev et al. 2017). Our comparative analysis suggests that differences in the regulatory signature (Arendt et al. 2016), rather than the overall gene expression patterns, are the key requirement for distinguishing cell types.

## Methods

### Genome assembly version

For consistency with previous FANTOM5 publications (The FANTOM Consortium and the RIKEN PMI and CLST (DGT) 2014; Lizio et al. 2017a,b), we used genome assemblies hg19 (human), mm9 (mouse), rn6 (rat), canFam3 (dog), and galGal5 (chicken) for our analysis. Previously, it was shown that 99.75% and 99.94% of CAGE peaks in human and mouse, respectively, could be converted unambiguously to the recent genome versions hg38 (human) and mm10 (mouse), with an expression correlation value larger than 0.99 both for human and mouse (Abugessaisa et al. 2017). For 231 (human) and 202 (mouse) miRNAs included in the comparative analysis shown in Supplemental Table S13, the genomic distance between each pre-miRNA and the corresponding pri-miRNA promoter (Supplemental Table S12) was identical between genome assembly versions for 216 (human) and 199 (mouse) miRNAs and differed by less than 10 base pairs for 227 (human) and 202 (mouse) miRNAs, suggesting that the genome assembly version used had minimal effects on the analysis results.

### Identification of orthologous genes

For each gene in mouse, rat, dog, and chicken defined in Ensembl (Zerbino et al. 2018) release 85, we retrieved the orthologous human gene, if defined, in an “ortholog_one2one” relationship with it in the Ensembl Compara multi-species database (Vilella et al. 2009). This yielded 16,217 (human-mouse), 15,486 (human-rat), 15,861 (human-dog), 11,950 (human-chicken), and 10,237 (in all five species) pairs of orthologous genes, of which 15,893 (human-mouse), 15,207 (human-rat), 15,482 (human-dog), 11,873 (human-chicken), and 10,208 (in all five species) were protein-coding. Using the most recent Ensembl release available for each genome assembly (release 75 for human genome assembly hg19, release 67 for mouse genome assembly mm9, release 85 for rat genome assembly rn6 and dog genome assembly canFam3, and release 92 for chicken genome assembly galGal5), we obtained the transcription start site for all transcripts associated with each gene, defined a ±500-bp promoter region around each transcription start site, and merged overlapping regions. Genes for which any of the associated regions had >10% unidentified nucleotides (N) in their genome sequence were removed from the analysis. The number of remaining orthologous protein-coding genes was 15,538 (human-mouse), 14,915 (human-rat), 13,759 (human-dog), 8696 (human-chicken), and 6561 (in all five species).

### Gene expression analysis

Gene expression quantitation is described in detail in the Supplemental Methods. Differential gene expression analysis was performed on the raw counts using DESeq2 (Love et al. 2014) version 1.22.1 with a threshold of 0.1 on the Benjamini–Hochberg adjusted *P*-value. PCA as well as all correlation calculations (except in Integrative Correlation Coefficient analysis, described below) were performed on variance-stabilized gene expression data generated as follows. First, we used DESeq2 (Love et al. 2014) version 1.22.1 to estimate, for each cell type in each species, the asymptotic dispersion of expression counts between replicates, and then calculated its average value *α* across cell types and species. Next, we calculated the total tag count for each sample, divided these totals by their median across samples to obtain the normalization factors, and divided the counts of each sample by the corresponding factor to obtain normalized count data *x*. We then applied the variance-stabilizing transformation (Love et al. 2014) to the normalized count data *x*

$$x\prime \;=\;\frac{2\;\mathrm{arcsinh}\left(\sqrt{\alpha x}\right)-\log\alpha -\log4\;}{\log2}$$

The variance-stabilized gene expression data *x*^′^ were averaged across replicates for each cell type and for each species.

For each pairwise comparison in Figure 2B, we calculated Pearson's correlation across cell types between each pair of orthologous genes. Next, we randomly permuted the gene pairings, calculated the correlation across cell types to find the background distribution, and performed the Mann–Whitney *U* test comparing the set of correlation values for pairs of orthologous genes to the set of correlation values for randomly permuted pairs. We also calculated a background distribution for pairs of orthologous genes after permuting the samples, as well as the cumulative distribution of correlation values for an uncorrelated bivariate normal distribution.

For the pairwise comparisons shown in Supplemental Figures S4–S11, we calculated Pearson's correlation between the two species for each cell type across orthologous genes. For Supplemental Figure S15, we calculated Pearson's correlation between the two species for each pair of orthologous genes across cell types.

### Promoter conservation analysis

Orthologous genomic regions of promoters across species were identified by applying liftOver (Hinrichs et al. 2006) on chain files downloaded from the University of California, Santa Cruz website (http://hgdownload.cse.ucsc.edu/downloads.html).

### Gene conservation analysis

For each gene in human, we identified the HomoloGene group of homologous genes to which it belonged in release 68 of the NCBI HomoloGene database (NCBI Resource Coordinators 2018). If the HomoloGene group included mammals only or vertebrates only, then the gene was classified as restricted to mammals or restricted to vertebrates, respectively. Alternatively, the gene was classified as conserved in bilateria if the HomoloGene group included bilateria in nonvertebrate lineages. To assess the statistical significance of the increase or decrease in conservation of expression in the three classes, the bilaterian, vertebrate, and mammalian class were represented by an equidistant indicator variable, and the maximum likelihood method was applied to fit a linear regression model under the Poisson distribution to the number of differentially expressed genes in each class. The corresponding *P*-value was calculated using the likelihood-ratio test. The overall *P*-value was calculated by combining the *P*-values for the pairwise comparisons using Fisher's method.

### Gene Ontology analysis

Gene Ontology annotations were downloaded on June 10, 2018 from the GOA database (Huntley et al. 2015). Statistical significance of over- or underrepresentation of a Gene Ontology term among differentially expressed genes was calculated using Fisher's exact test, where an expression-matched background was created by selecting the 10 closest genes in expression in human for each differentially expressed gene. The overall *P*-value was calculated by combining the *P*-values for the pairwise comparisons using Fisher's method.

### RNA-seq expression data analysis

Accession numbers for ENCODE (The ENCODE Project Consortium 2012) and endometrial stromal fibroblast (Kin et al. 2016) RNA-seq gene expression data are provided in the Supplemental Methods. Gene conservation and Gene Ontology analysis of these data sets were performed as described above.

### Integrative Correlation Coefficient analysis

Integrative Correlation Coefficient analysis (Parmigiani et al. 2004) ranks genes based on the degree to which their expression profiles are comparable between data sets.

For human and mouse separately, we constructed a CAGE expression matrix (normalized to t.p.m.) for the 15,538 genes in common between human and mouse, averaging biological replicates by taking the median, and performed quantile normalization separately for each expression matrix. Next, we calculated the correlation between each pair of genes, again for human and mouse separately, across cell types to obtain one correlation matrix for human and one correlation matrix for mouse. We then calculated Pearson's correlation between human and mouse for corresponding rows in these two correlation matrices to obtain the correlation-of-correlations, or integrative correlation coefficient, for each gene. The null distribution was obtained by randomly permuting samples 10,000 times, as described previously (Parmigiani et al. 2004), using MergeMaid (Cope et al. 2004) version 2.56.0. Analysis of Functional Annotation (AFA) (Ross et al. 2011; Kortenhorst et al. 2013; Marchionni et al. 2017) was conducted by performing a one-sided Wilcoxon rank-sum test to compare the integrative correlation coefficient values of genes in each cellular component (CC) and biological process (BP) Gene Ontology category (extracted using the “org.Hs.eg.db” R/Bioconductor package version 3.8.2), requiring at least 10 genes, to those of remaining genes, using the Benjamini–Hochberg multiple testing correction method. All analyses were performed using the R/Bioconductor “RTopper” package (version 1.30.0) (Tyekucheva et al. 2011).

### Multiple genome alignment, TFBS prediction, and motif activity analysis

The 100-way multiple genome alignment of human genome assembly hg19 against 99 vertebrate species and the 30-way multiple genome alignment of the mouse genome assembly mm9 against 29 vertebrate species were downloaded from the University of California, Santa Cruz website (http://hgdownload.cse.ucsc.edu/downloads.html), the species in the 30-way mouse alignment being a subset of the species in the 100-way human alignment. For the same set of 30 species, we performed pairwise genome alignments of the rat, dog, and chicken genome against each of the 29 remaining species for the genome assemblies listed in Supplemental Table S14 (see Supplemental Methods for details). Pairwise alignments were merged into a multiple genome alignment using MULTIZ (Blanchette et al. 2004) version 11.2 using the phylogenetic tree of the 30 species extracted from the 191-way phylogenetic tree in 191way.nh distributed as part of the UCSC Genome Browser bioinformatics utilities (Kuhn et al. 2013) release 366 (June 5, 2018). Genome-wide TFBS predictions and motif activity analysis were performed as described previously (Arner et al. 2015), with minor modifications as described in the Supplemental Methods. The multiple genome alignment files, genome-wide locations and scores of predicted TFBSs, and motif activity scripts are available at http://fantom.gsc.riken.jp/5/suppl/Alam_et_al_2020/; motif activity scripts are also included in the Supplemental Code.

### Enhancer identification

The previously calculated set of permissive enhancers (Arner et al. 2015) was used for human (65,423 enhancers) and mouse (44,459 enhancers). For rat, dog, and chicken, we first created a mask for all ±500-bp windows around the 5′ end of transcripts in the NCBI Entrez Gene database (Brown et al. 2015), downloaded on November 13, 2017, as well as all windows within 200 bp of exons defined in the same database. We then applied the bidir_enhancers script (Andersson et al. 2014) to all FANTOM5 CAGE libraries in rat, dog (Lizio et al. 2017b), and chicken (Lizio et al. 2017a) using the calculated mask, resulting in 9372 (rat), 10,649 (dog), and 44,625 (chicken) enhancers.

### MicroRNA analysis

Short RNA libraries were produced, sequenced, and processed as described previously (De Rie et al. 2017) using the same RNA samples as used for CAGE expression profiling (Lizio et al. 2017a,b). Short RNA libraries not described previously are listed with their matching CAGE library in Supplemental Table S1. Annotation of miRNAs, candidate novel miRNA prediction, and miRNA promoter identification were performed as described in the Supplemental Methods. Orthologous miRNAs were identified by performing global alignment of mature miRNA sequences between species, followed by manual curation. The evolutionary age of miRNAs was established based on the set of species in which miRNAs of each family were annotated in miRBase release 21 (Kozomara and Griffiths-Jones 2014).

## Data access

All raw and processed sequencing data generated in this study have been submitted to the DNA Data Bank of Japan (DDBJ; https://www.ddbj.nig.ac.jp/) under accession number DRA008211. All custom scripts generated in this study are available as Supplemental Code.

## Competing interest statement

The authors declare no competing interests.

## Supplementary Material

Supplemental Material
