# Supplementary material for: Comparative transcriptomics of primary cells in vertebrates
Source: Genome Res. 2020 Jul;30(7):951–61. doi: 10.1101/gr.255679.119 (PMC7397866; doi:10.1101/gr.255679.119)
Supplement: Supplemental Material [file supp_30_7_951__index.html]

Comparative transcriptomics of primary cells in vertebrates — Supplemental Material 

# Comparative transcriptomics of primary cells in vertebrates

## Supplemental Material

- Supplemental\_figure.pdf
- Supplemental\_Table\_S1.xlsx
- Supplemental\_Table\_S2.xlsx
- Supplemental\_Table\_S3.xlsx
- Supplemental\_Table\_S4.xlsx
- Supplemental\_Table\_S5.xlsx
- Supplemental\_Table\_S6.xlsx
- Supplemental\_Table\_S7.xlsx
- Supplemental\_Table\_S8.xlsx
- Supplemental\_Table\_S9.xlsx
- Supplemental\_Table\_S10.xlsx
- Supplemental\_Table\_S11.xlsx
- Supplemental\_Table\_S12.xlsx
- Supplemental\_Table\_S13.xlsx
- Supplemental\_Table\_S14.xlsx
- Supplemental\_code.zip
- Supplemental\_Methods.docx
