# Supplementary material for: Comparative transcriptomics of primary cells in vertebrates
Source: Genome Res. 2020 Jul;30(7):951–61. doi: 10.1101/gr.255679.119 (PMC7397866; doi:10.1101/gr.255679.119)
Supplement: Supplemental Material [file supp_gr.255679.119_Supplemental_figure.pdf]

Supplemental Figures

This file contains the following Supplemental Figures:

Supplemental Figure S1	Promoter analysis of differentially expressed genes compared to an expression-matched background.
Supplemental Figure S2	Conservation analysis of differentially expressed genes compared to an expression-matched background.
Supplemental Figure S3	Integrative Correlation Analysis.
Supplemental Figure S4	Gene expression correlation of human and mouse samples.
Supplemental Figure S5	Gene expression correlation of human, mouse, rat, dog, and chicken samples.
Supplemental Figure S6	Phylogenetic analysis of expression divergence.
Supplemental Figure S7	Promoter analysis of gene expression correlations.
Supplemental Figure S8	Promoter analysis of gene expression correlations compared to an expression-matched background.
Supplemental Figure S9	Conservation analysis of gene expression correlations.
Supplemental Figure S10	Conservation analysis of gene expression correlations compared to an expression-matched background.
Supplemental Figure S11	Gene Ontology analysis of gene expression correlations.
Supplemental Figure S12	Analysis of RNA-Seq expression data for endometrial stromal fibroblast primary cells (Kin et al., 2016).
Supplemental Figure S13	Comparative analysis of RNA-Seq expression data for 12 matching tissues in human and mouse (The ENCODE Project Consortium, 2012).
Supplemental Figure S14	Evaluation of predicted transcription factor binding sites.
Supplemental Figure S15	Pearson's correlation across cell types between orthologous genes, as a function of TFBS conservation in their promoter regions.
Supplemental Figure S16	Cumulative distribution of the Pearson's correlation *r* across cell types in motif activity between promoters and enhancers in human, mouse, rat, dog, and chicken.
Supplemental Figure S17	Comparison of CAGE expression levels of the pri-miRNA to the mature miRNA expression levels measured by sRNA sequencing in aortic smooth muscle cells in human, mouse, rat, dog, and chicken.
Supplemental Figure S18	Comparison of differential expression analysis of pri-miRNAs and mature miRNAs.

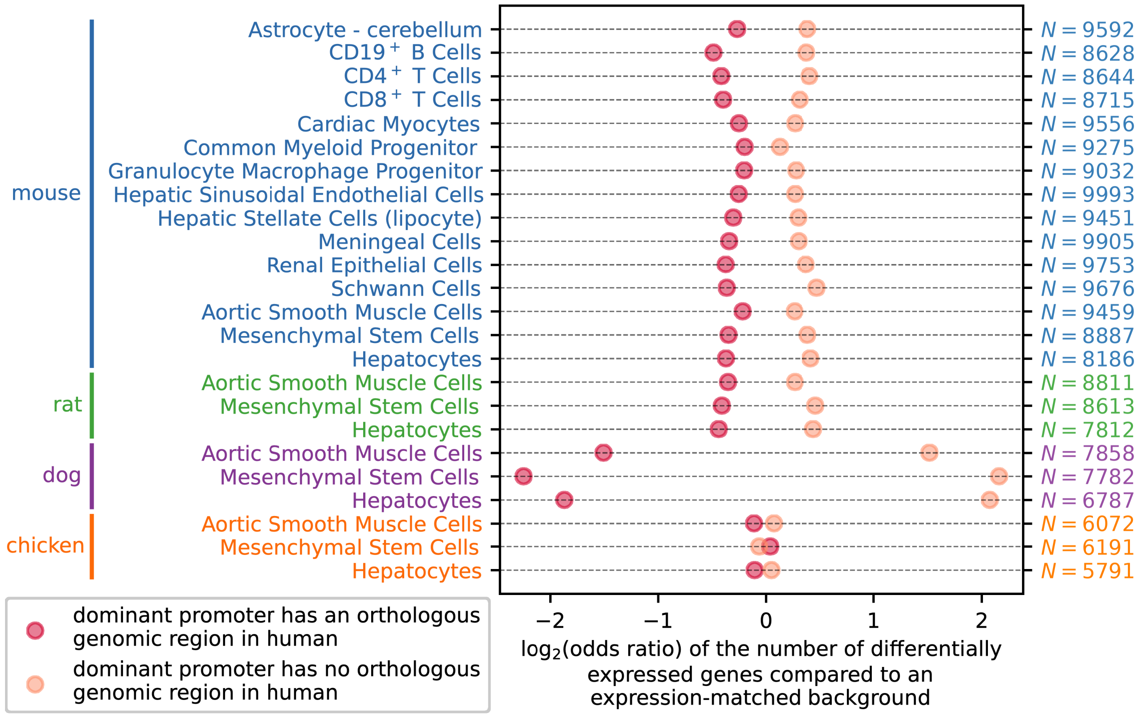

p-value, Fisher's exact test

● (orthologous)	● (no orthologous)
5.7×10^{-16}	6.7×10^{-5}
3.1×10^{-43}	0.0002
6.6×10^{-33}	3.9×10^{-5}
2.1×10^{-29}	0.0019
3.4×10^{-14}	0.0047
2.8×10^{-9}	0.17
8.6×10^{-9}	0.0048
1.4×10^{-9}	0.029
9.0×10^{-18}	0.0031
1.1×10^{-25}	0.0012
3.2×10^{-17}	0.0035
9.6×10^{-27}	1.5×10^{-6}
8.8×10^{-11}	0.0061
6.4×10^{-22}	0.00026
2.8×10^{-25}	5.8×10^{-5}
1.6×10^{-19}	0.011
4.0×10^{-30}	1.3×10^{-6}
5.6×10^{-28}	6.2×10^{-5}
$< 1 \times 10^{-100}$	3.2×10^{-30}
$< 1 \times 10^{-100}$	2.1×10^{-82}
$< 1 \times 10^{-100}$	2.5×10^{-49}
0.085	0.26
0.47	0.26
0.072	0.38

Supplemental Figure S1.

Promoter analysis of differentially expressed genes compared to an expression-matched background. Genes were divided into two categories depending on whether the genomic region of the dominant promoter of the gene had an orthologous genomic region in the human genome. The enrichment was calculated by comparing the number of differentially expressed genes in each of the two categories to the number of differentially expressed genes in an expression-matched background, with the $\log_2$(odds ratio) shown on the horizontal axis of the left panel and *p*-value calculated using Fisher's exact test for each comparison in the right panel. *N* is the total number of expressed genes in each cell type.

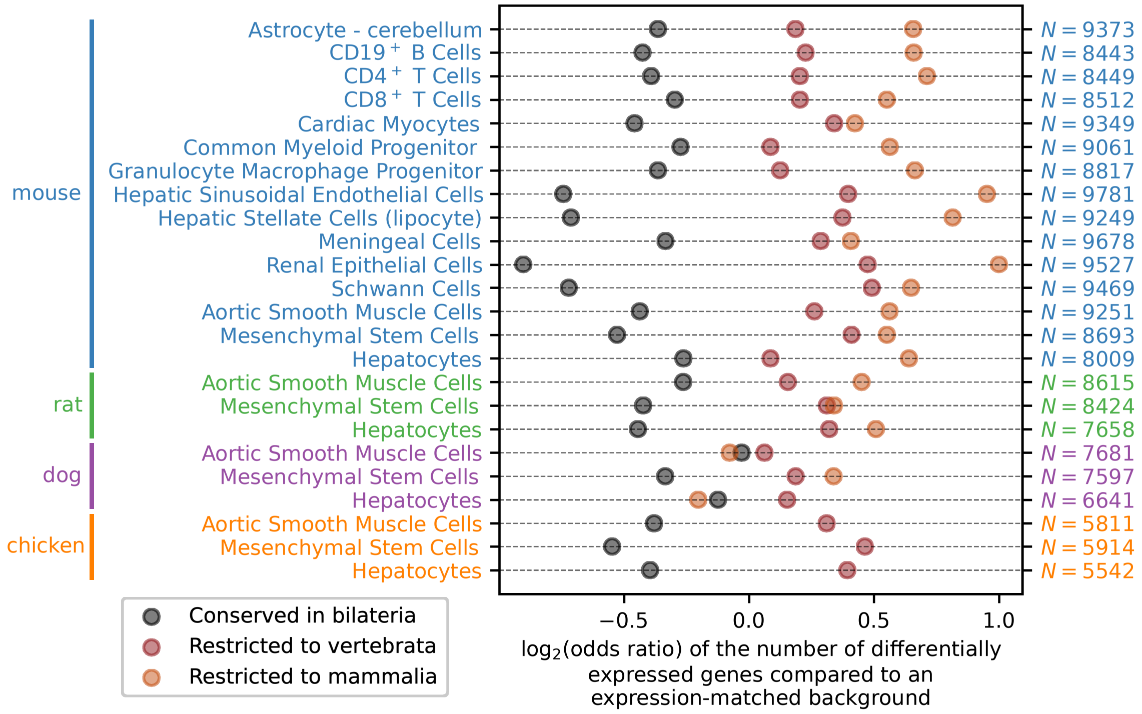

p-value, Fisher's exact test

Cell type	Conserved in bilateria	Restricted to vertebrata	Restricted to mammalia
mouse Astrocyte - cerebellum	1.6×10^{-14}	4.8×10^{-5}	2.1×10^{-8}
mouse CD19+ B Cells	8.9×10^{-19}	5.8×10^{-6}	1.8×10^{-7}
mouse CD4+ T Cells	2.6×10^{-16}	3.3×10^{-5}	9.1×10^{-9}
mouse CD8+ T Cells	7.7×10^{-10}	4.0×10^{-5}	1.7×10^{-5}
mouse Cardiac Myocytes	4.4×10^{-22}	1.3×10^{-13}	0.00037
mouse Common Myeloid Progenitor	4.6×10^{-9}	0.064	1.5×10^{-6}
mouse Granulocyte Macrophage Progenitor	1.2×10^{-13}	0.011	2.3×10^{-8}
mouse Hepatic Sinusoidal Endothelial Cells	5.5×10^{-33}	1.0×10^{-11}	1.4×10^{-12}
mouse Hepatic Stellate Cells (lipocyte)	8.9×10^{-45}	6.1×10^{-14}	1.1×10^{-11}
mouse Meningeal Cells	9.2×10^{-13}	2.0×10^{-10}	0.00047
mouse Renal Epithelial Cells	6.1×10^{-40}	1.4×10^{-14}	1.3×10^{-12}
mouse Schwann Cells	4.2×10^{-48}	1.0×10^{-25}	7.1×10^{-8}
mouse Aortic Smooth Muscle Cells	2.1×10^{-20}	1.7×10^{-8}	2.6×10^{-6}
mouse Mesenchymal Stem Cells	2.1×10^{-26}	4.3×10^{-16}	3.3×10^{-5}
mouse Hepatocytes	5.6×10^{-8}	0.092	1.7×10^{-6}
rat Aortic Smooth Muscle Cells	5.9×10^{-7}	0.0036	0.002
rat Mesenchymal Stem Cells	4.3×10^{-18}	3.7×10^{-10}	0.0098
rat Hepatocytes	3.7×10^{-17}	1.2×10^{-8}	0.00098
dog Aortic Smooth Muscle Cells	0.65	0.36	0.67
dog Mesenchymal Stem Cells	5.5×10^{-10}	0.001	0.021
dog Hepatocytes	0.051	0.031	0.25
chicken Aortic Smooth Muscle Cells	3.5×10^{-9}	3.2×10^{-6}	
chicken Mesenchymal Stem Cells	8.3×10^{-23}	1.0×10^{-16}	
chicken Hepatocytes	6.9×10^{-12}	6.1×10^{-11}	

Supplemental Figure S2.

Conservation analysis of differentially expressed genes compared to an expression-matched background. Genes were divided into three categories based on the age of the most recent common ancestor. The enrichment was calculated by comparing the number of differentially expressed genes to the number of differentially expressed genes in an expression-matched background, with the $\log_2$(odds ratio) shown on the horizontal axis of the left panel and p-value calculated using Fisher's exact test for each comparison in the right panel. N is the total number of expressed genes in each cell type with an annotation in the NCBI HomoloGene database.

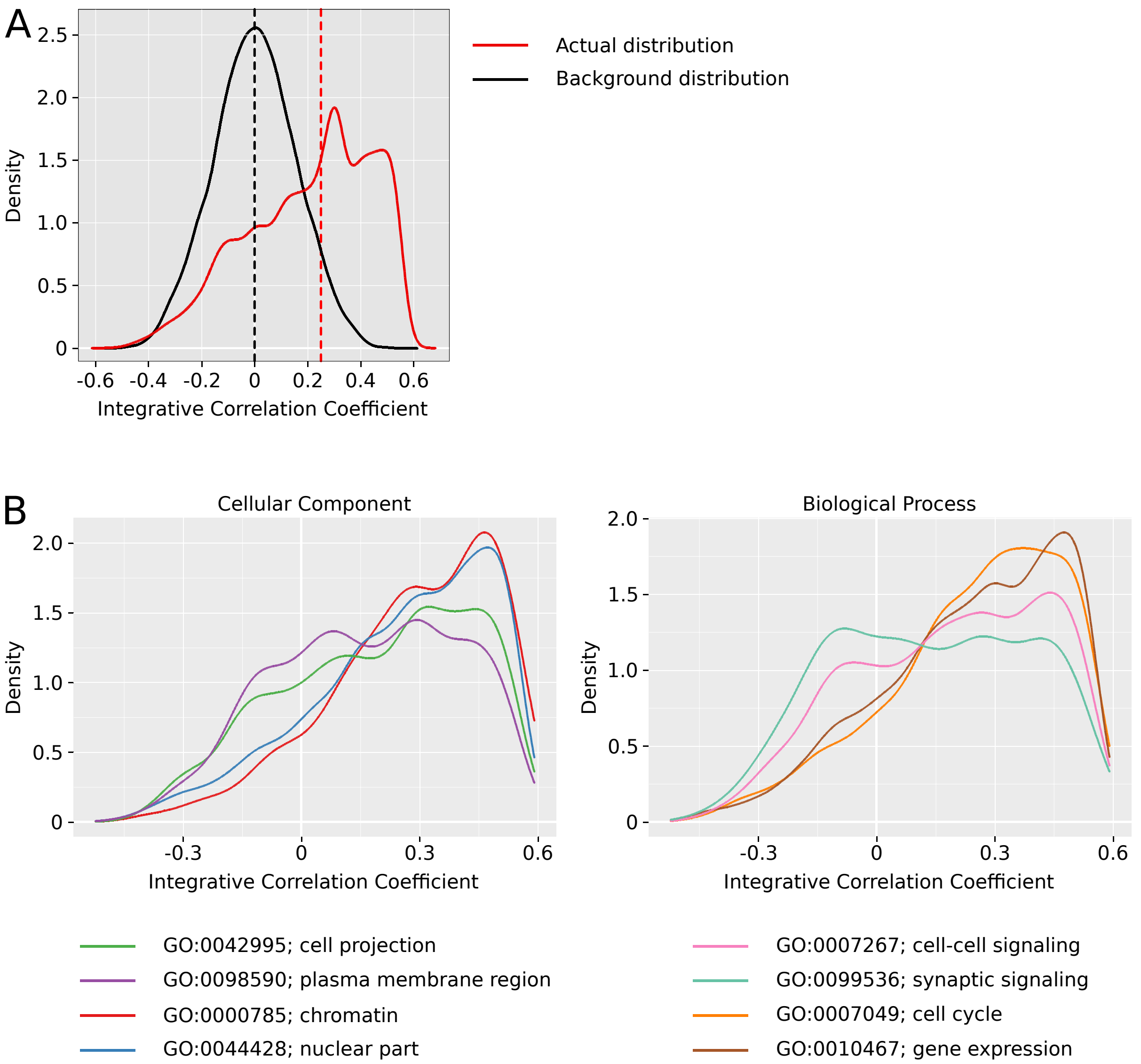

Supplemental Figure S3.

Integrative Correlation Analysis. (*A*) Distribution of the integrative correlation coefficient computed for 15,538 expressed genes in common between human and mouse. The actual distribution is shown in red, while the null distribution obtained by permutation is depicted in black. Dashed lines indicate the median of each distribution. (*B*) Examples the distribution of the integrative correlation coefficient between human and mouse for genes belonging to specific Gene Ontology terms identified as significant (FDR < 0.05) by Analysis of Functional Annotation. The distribution for Gene Ontology terms associated the key cellular processes and components (chromatin, nuclear part, cell cycle, and gene expression) are shifted to right suggesting a higher degree of conservation, while processes related to cell-to cell interaction (cell-cell and synaptic signaling, cell projection and plasma membrane region) are skewed to the left, suggesting a lesser degree of conservation across species.

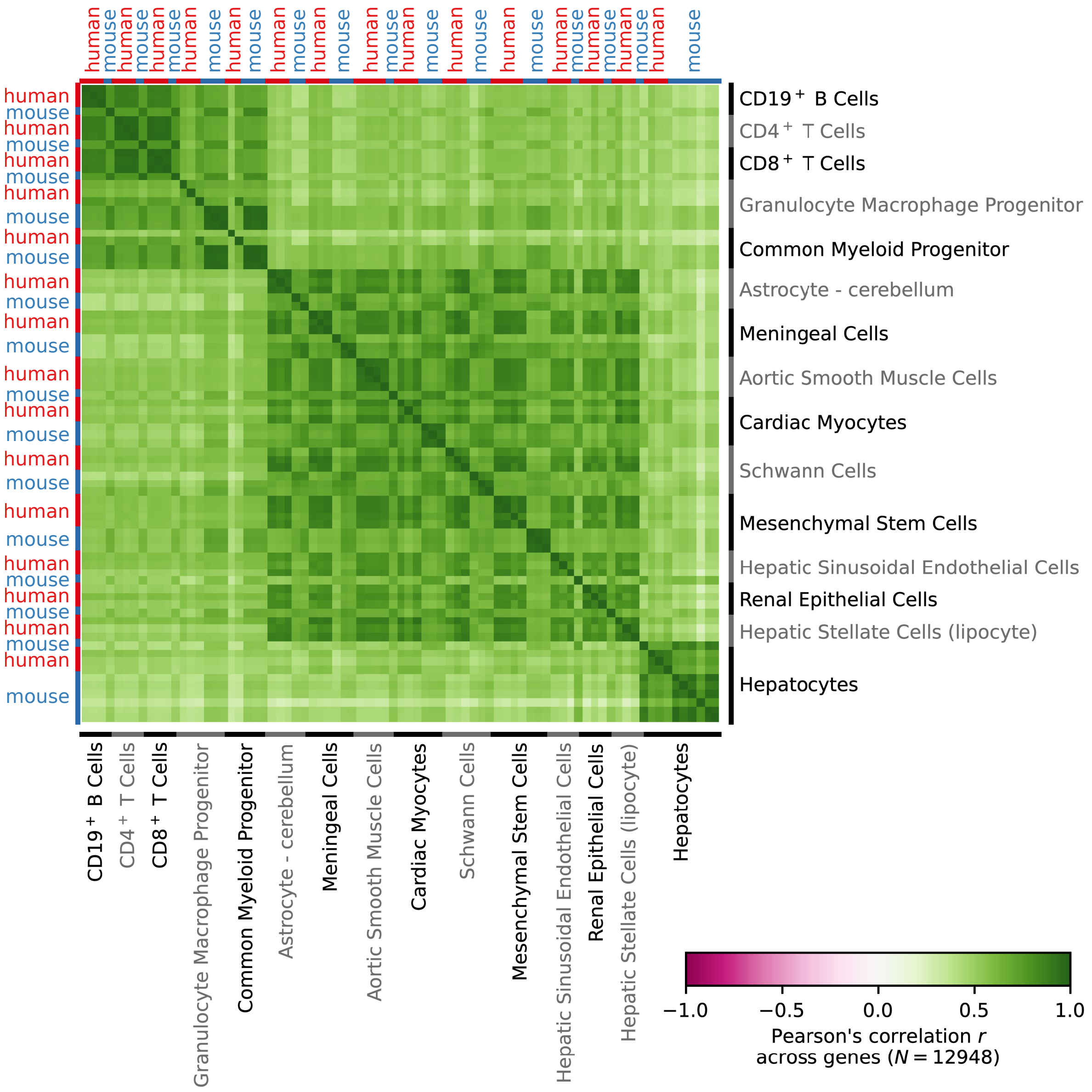

Supplemental Figure S4.

Gene expression correlation of human and mouse samples. The heatmap visualizes the value of Pearson's correlation coefficient across genes between all samples for cell types with CAGE expression data in both human and mouse.

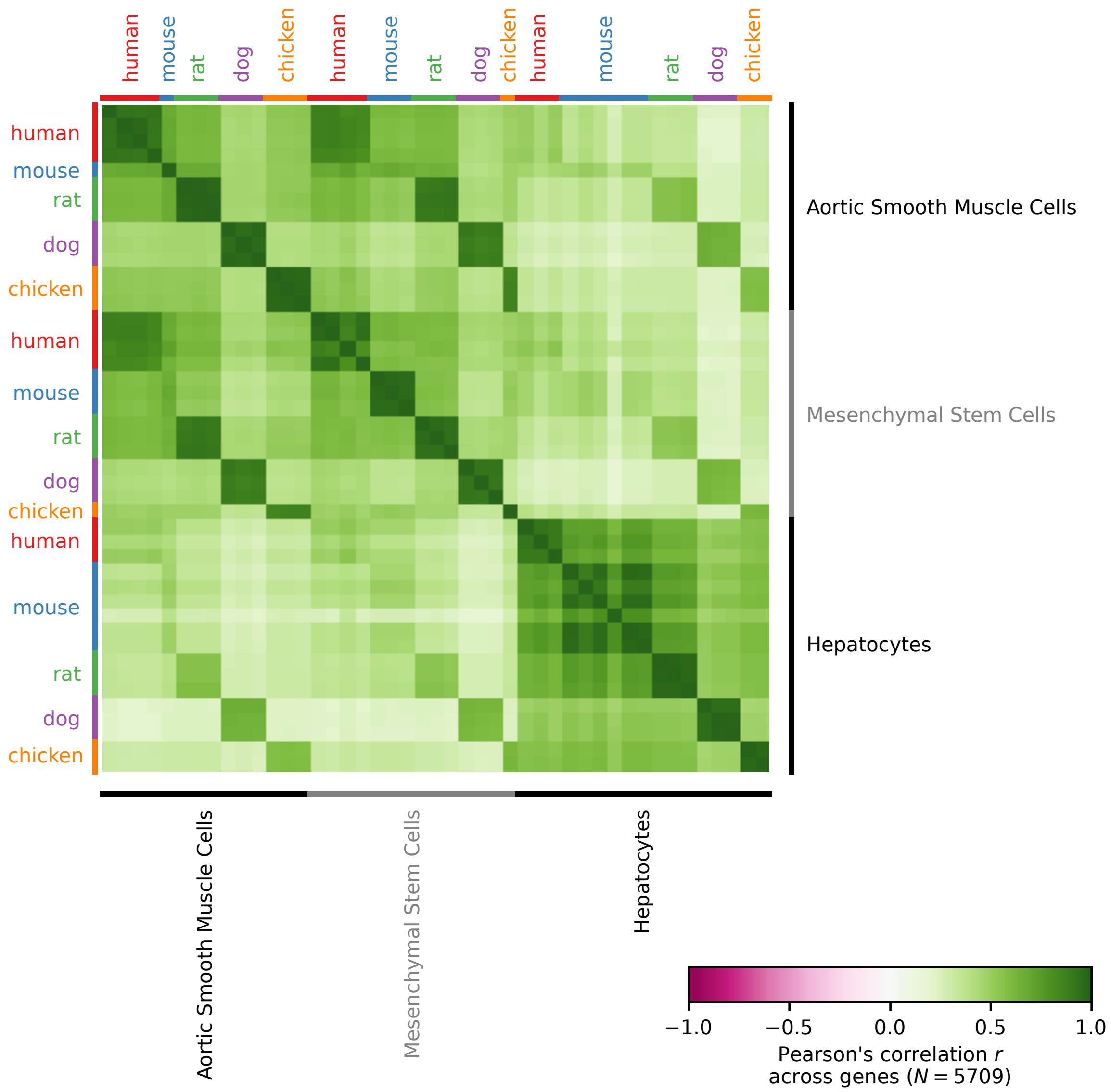

Supplemental Figure S5.

Gene expression correlation of human, mouse, rat, dog, and chicken samples. The heatmap visualizes the value of Pearson's correlation coefficient across genes between all samples for cell types with CAGE expression data in all five species.

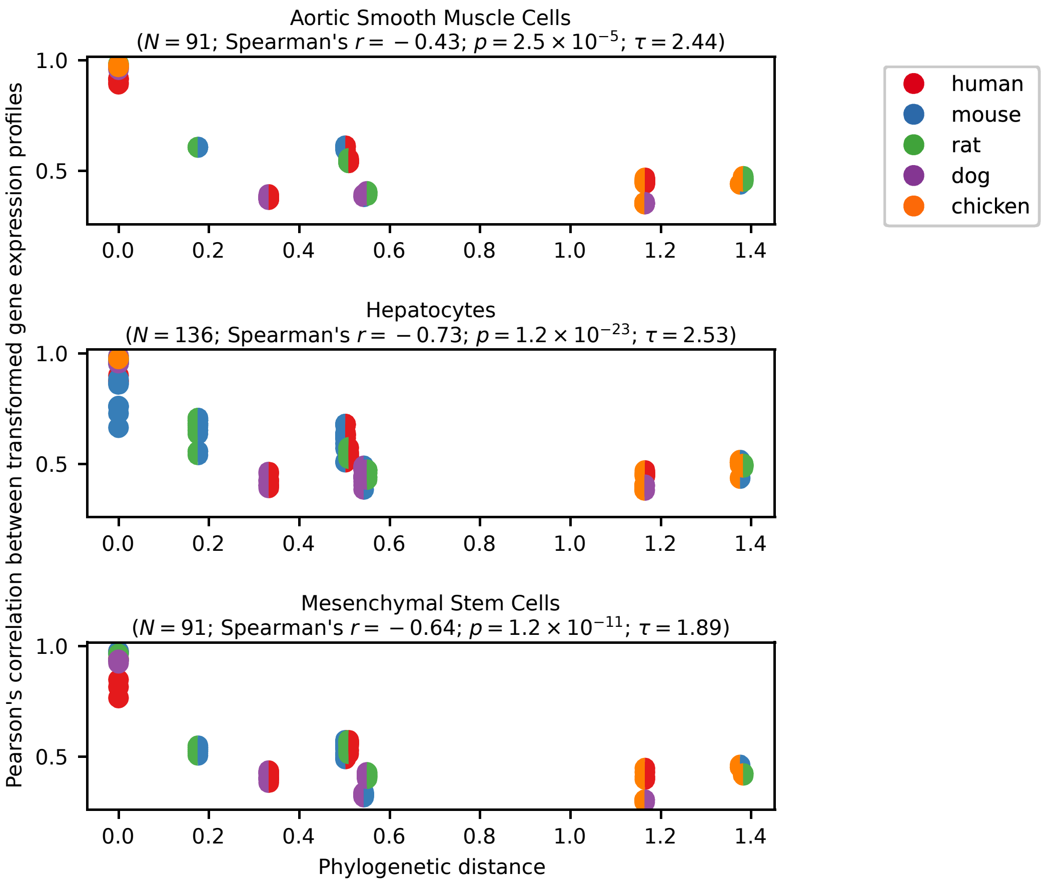

Supplemental Figure S6.

Phylogenetic analysis of expression divergence. The value of Pearson's correlation *r* across genes is shown as a function of phylogenetic distance between species. Phylogenetic distances were calculated as the sum of branch lengths (which are proportional to the average number of substitutions per site in the genome) in the phylogenetic tree provided by UCSC (Miller et al. 2007). The decay time τ was estimated by linear regression of the natural logarithm of *r* against the phylogenetic distance.

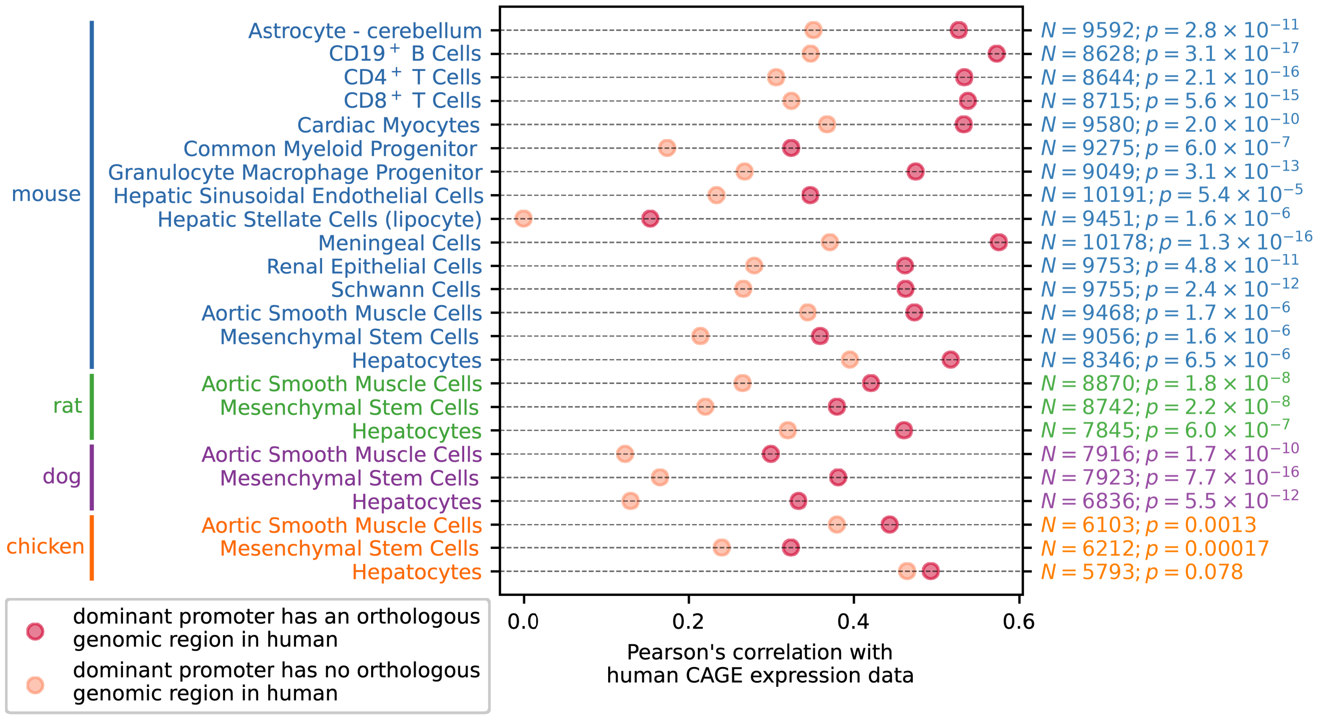

Supplemental Figure S7.

Promoter analysis of gene expression correlations. Pearson's correlation in each cell type and species was calculated separately for genes for which the dominant promoter had an orthologous genomic region in the human genome, and for genes for which the dominant promoter did not have an orthologous genomic region in the human genome. The one-sided p-value shown on the right was calculated by applying the Fisher Z-transformation to each correlation value and performing a paired Z-test. N is the number of expressed genes in each cell type.

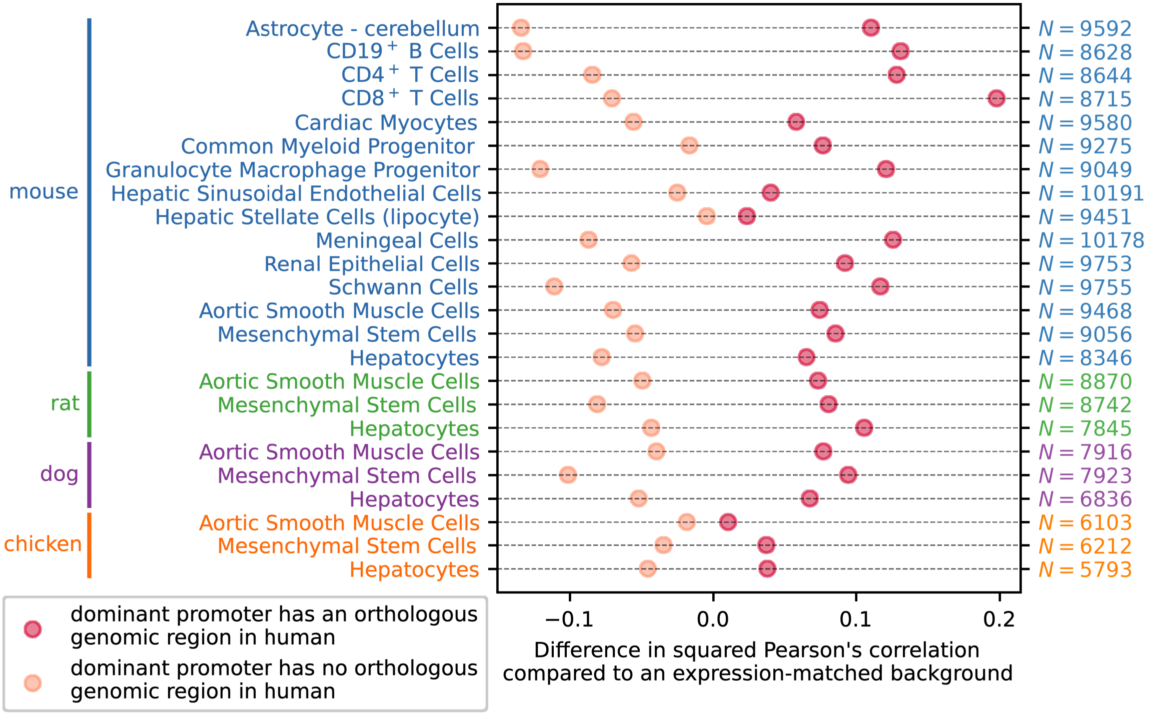

p-value calculated using the Fisher *Z* transformation

1.7×10^{-5}	3.1×10^{-23}
3.3×10^{-5}	3.0×10^{-27}
0.0035	5.8×10^{-28}
0.016	2.2×10^{-72}
0.064	4.1×10^{-7}
0.32	1.1×10^{-26}
1.3×10^{-5}	1.8×10^{-30}
0.23	1.9×10^{-6}
0.13	4.8×10^{-24}
0.0039	1.9×10^{-29}
0.025	2.0×10^{-19}
3.7×10^{-5}	5.0×10^{-32}
0.018	4.1×10^{-12}
0.015	1.3×10^{-25}
0.02	7.2×10^{-8}
0.036	8.1×10^{-13}
0.00032	1.3×10^{-18}
0.12	9.9×10^{-21}
0.0024	2.7×10^{-29}
3.5×10^{-8}	9.1×10^{-23}
0.0007	6.2×10^{-13}
0.28	0.56
0.0068	0.0062
0.019	0.052

Supplemental Figure S8.

Promoter analysis of gene expression correlations compared to an expression-matched background. Difference in squared Pearson's correlation r^2 in each cell type and species, relative to an expression-matched background, was calculated for genes for which the dominant promoter had an orthologous genomic region in the human genome, and for genes for which the dominant promoter did not have an orthologous genomic region in the human genome. The one-sided *p*-value shown on the right was calculated by applying the Fisher *Z*-transformation to each correlation value and performing a paired *Z*-test relative to the expression-matched background. *N* is the number of expressed genes in each cell type.

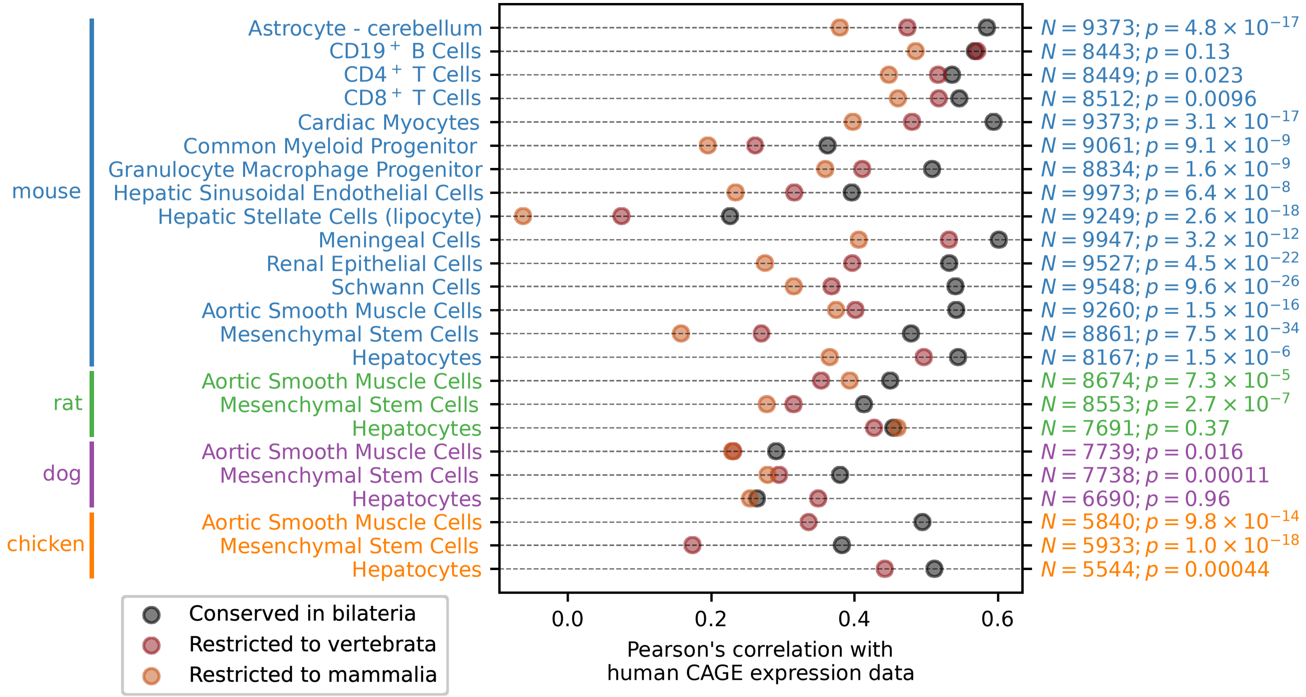

Supplemental Figure S9.

Conservation analysis of gene expression correlations. Pearson's correlation was calculated in each cell type and species across orthologous genes for three categories of genes defined by the age of their most recent common ancestor. The Fisher *Z*-transformation was applied to each correlation value to obtain *Z*-scores. The one-sided *p*-value of a linear regression model of the *Z*-scores against the evolutionary age category is shown on the right, together with the number *N* of expressed genes in each cell type with an annotation in the NCBI HomoloGene database.

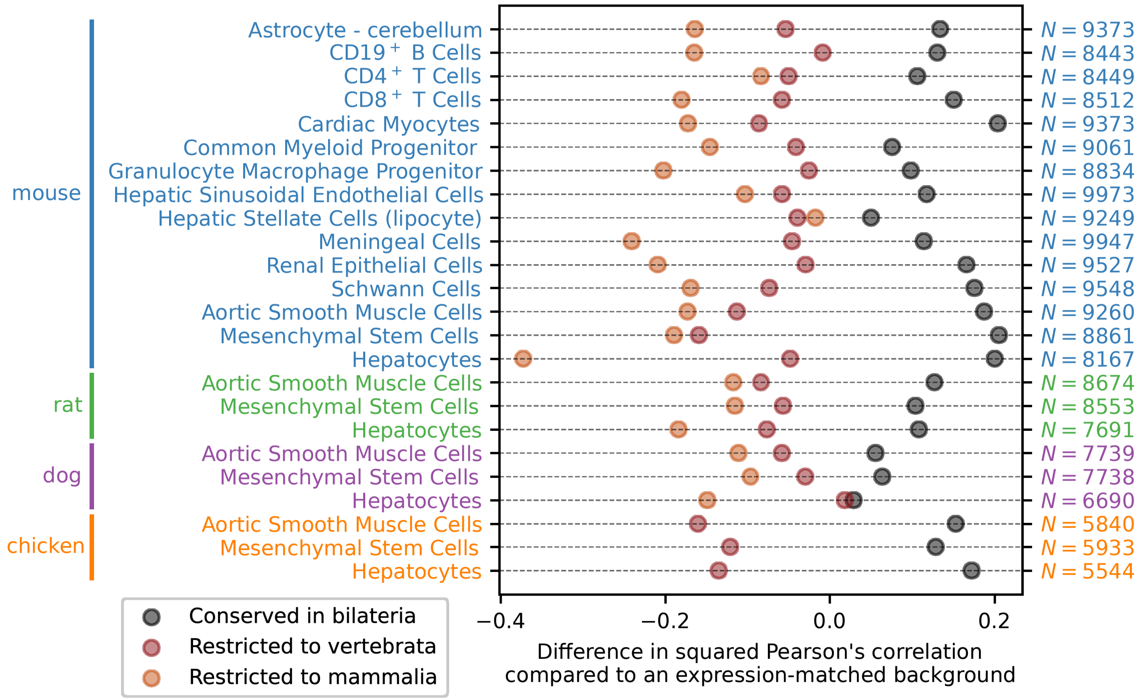

p-value calculated using the Fisher Z transformation

3.5×10^{-5}	0.00075	3.3×10^{-16}
0.00012	0.63	4.5×10^{-15}
0.048	0.0044	9.3×10^{-11}
3.1×10^{-5}	0.0009	8.2×10^{-21}
2.3×10^{-5}	9.3×10^{-8}	1.5×10^{-37}
1.7×10^{-6}	0.00053	1.7×10^{-10}
3.4×10^{-7}	0.11	4.5×10^{-10}
0.00039	5.1×10^{-6}	1.3×10^{-24}
0.00014	8.0×10^{-11}	6.5×10^{-20}
1.8×10^{-9}	0.0035	1.9×10^{-12}
7.5×10^{-9}	0.044	3.7×10^{-28}
6.3×10^{-6}	3.1×10^{-7}	4.8×10^{-31}
1.8×10^{-5}	3.1×10^{-13}	3.5×10^{-35}
9.1×10^{-10}	1.9×10^{-29}	1.5×10^{-61}
1.7×10^{-17}	0.0071	2.8×10^{-38}
0.0045	2.7×10^{-8}	8.4×10^{-20}
0.0014	4.5×10^{-5}	5.4×10^{-15}
9.4×10^{-5}	1.6×10^{-5}	6.7×10^{-13}
0.00093	2.5×10^{-6}	5.6×10^{-8}
0.0071	0.029	3.9×10^{-6}
0.00031	0.24	0.0067
	1.8×10^{-18}	1.3×10^{-18}
	1.4×10^{-19}	3.8×10^{-25}
	5.2×10^{-11}	2.2×10^{-22}

Supplemental Figure S10.

Conservation analysis of gene expression correlations compared to an expression-matched background. Difference in squared Pearson's correlation r^2 in each cell type and species, relative to an expression-matched background, was calculated for three categories of genes defined by the age of their most recent common ancestor. The one-sided p-value shown on the right was calculated by applying the Fisher Z-transformation to each correlation value and performing a paired Z-test relative to the expression-matched background. N is the number of expressed genes in each cell type.

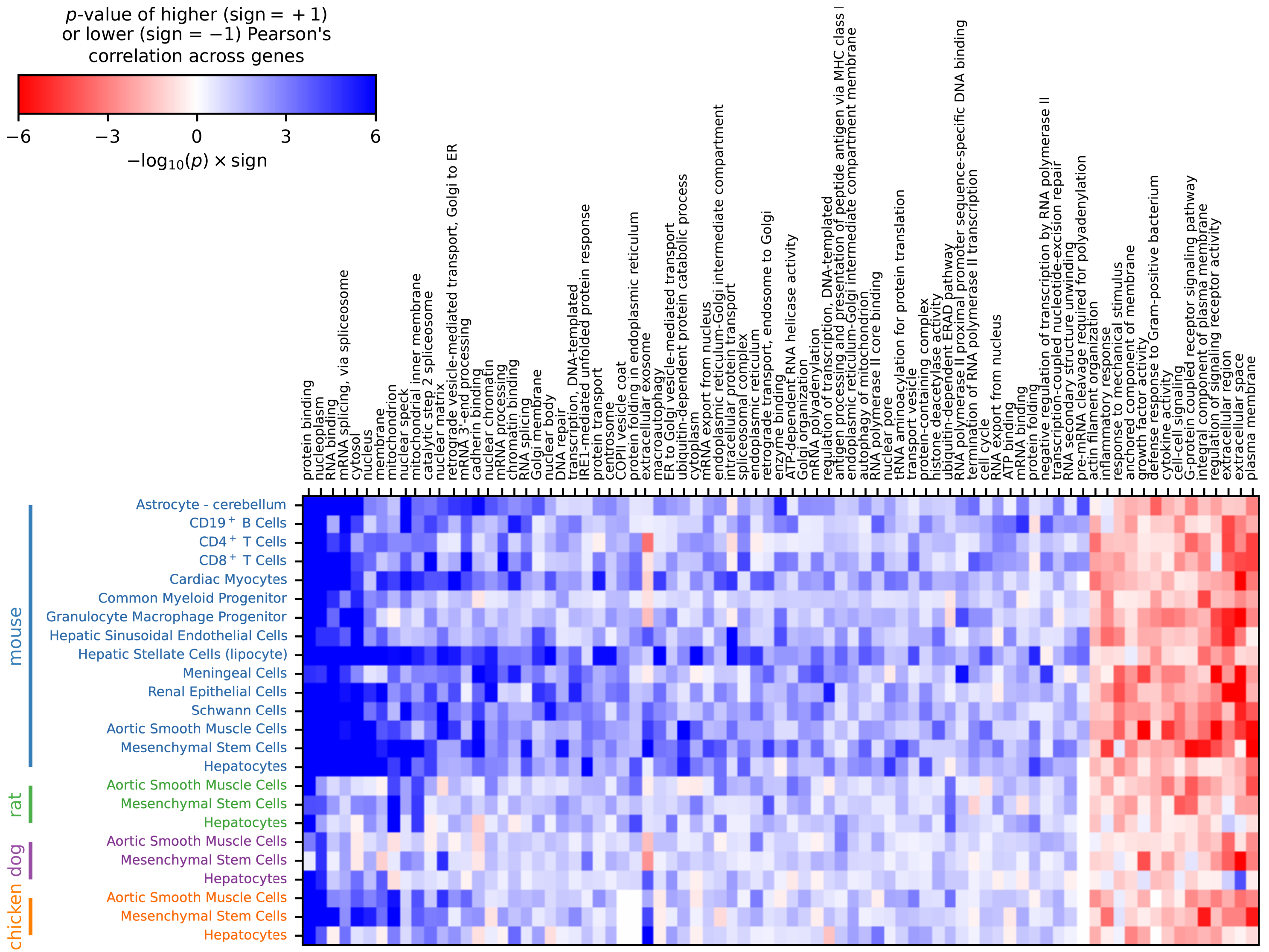

Supplemental Figure S11.

Gene Ontology analysis of gene expression correlations. The *p*-value of each Gene Ontology category in each cell type and species was calculated by applying the Fisher *Z*-transformation to the correlation value and performing a paired *Z*-test relative to the expression-matched background, with a higher or lower correlation value shown in blue and red, respectively.

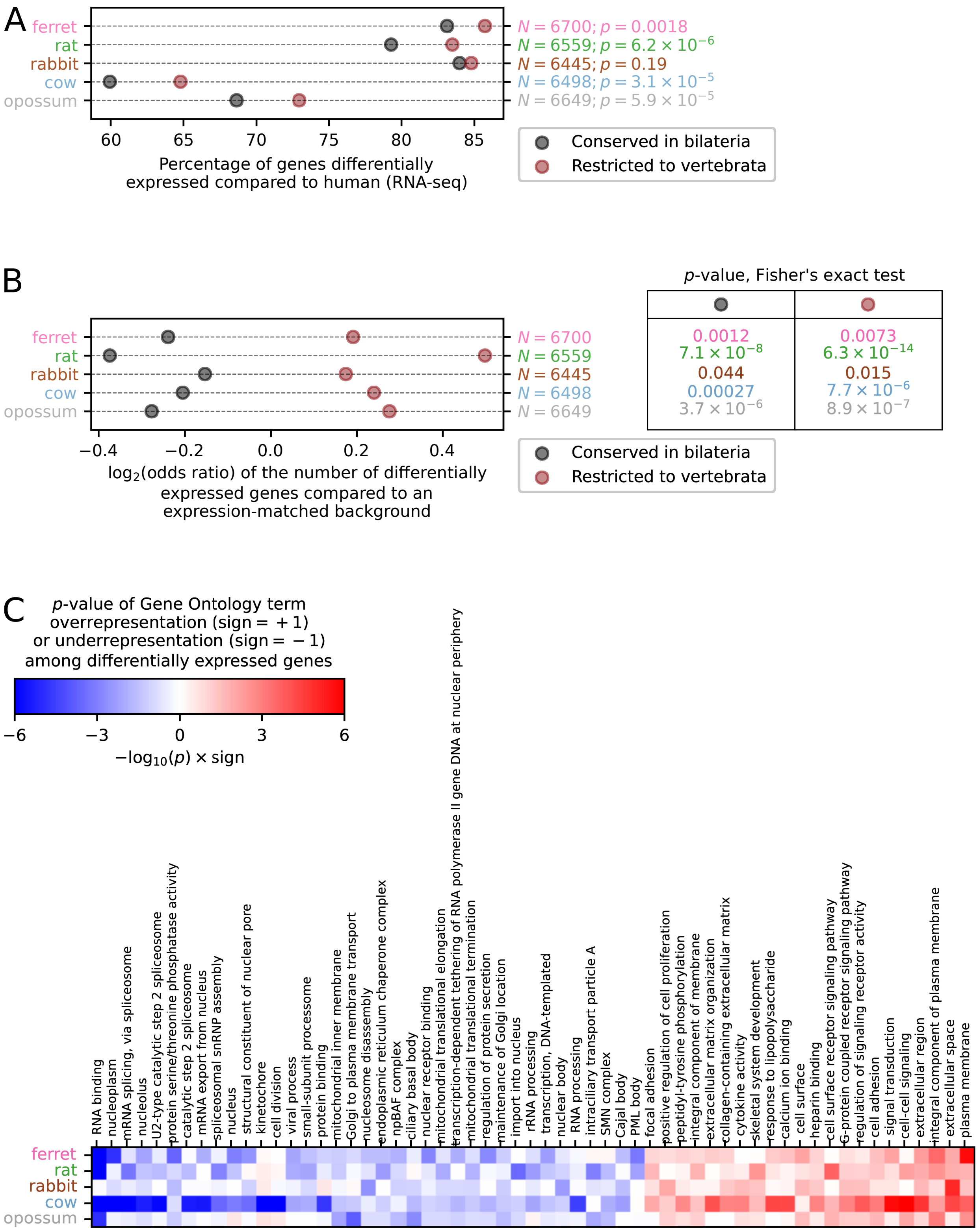
A
ferret
rat
rabbit
cow
opossum
N = 6700; p = 0.0018
N = 6559; p = 6.2 × 10^−6
N = 6445; p = 0.19
N = 6498; p = 3.1 × 10^−5
N = 6649; p = 5.9 × 10^−5
60 65 70 75 80 85
Percentage of genes differentially expressed compared to human (RNA-seq)
Conserved in bilateria
Restricted to vertebrata
B
p-value, Fisher's exact test
0.0012 0.0073
7.1 × 10^−8 6.3 × 10^−14
0.044 0.015
0.00027 7.7 × 10^−6
3.7 × 10^−6 8.9 × 10^−7
ferret
rat
rabbit
cow
opossum
N = 6700
N = 6559
N = 6445
N = 6498
N = 6649
−0.4 −0.2 0.0 0.2 0.4
log2(odds ratio) of the number of differentially expressed genes compared to an expression-matched background
Conserved in bilateria
Restricted to vertebrata
C
p-value of Gene Ontology term overrepresentation (sign = +1) or underrepresentation (sign = −1) among differentially expressed genes
−6 −3 0 3 6
−log10(p) × sign
RNA binding
nucleoplasm
mRNA splicing, via spliceosome
nucleolus
U2-type catalytic step 2 spliceosome
protein serine/threonine phosphatase activity
catalytic step 2 spliceosome
mRNA export from nucleus
spliceosomal snRNP assembly
nucleus
structural constituent of nuclear pore
kinetochore
cell division
viral process
small-subunit processome
protein binding
mitochondrial inner membrane
Golgi to plasma membrane transport
nucleosome disassembly
endoplasmic reticulum chaperone complex
npBAF complex
ciliary basal body
nuclear receptor binding
mitochondrial translational elongation
transcription-dependent tethering of RNA polymerase II gene DNA at nuclear periphery
mitochondrial translational termination
regulation of protein secretion
maintenance of Golgi location
import into nucleus
rRNA processing
transcription, DNA-templated
nuclear body
RNA processing
intraciliary transport particle A
SMN complex
Cajal body
PML body
focal adhesion
positive regulation of cell proliferation
peptidyl-tyrosine phosphorylation
integral component of membrane
extracellular matrix organization
collagen-containing extracellular matrix
cytokine activity
skeletal system development
response to lipopolysaccharide
calcium ion binding
cell surface
heparin binding
cell surface receptor signaling pathway
G-protein coupled receptor signaling pathway
regulation of signaling receptor activity
cell adhesion
signal transduction
cell-cell signaling
extracellular region
integral component of plasma membrane
extracellular space
plasma membrane
ferret
rat
rabbit
cow
opossum

Supplemental Figure S12 (previous page).

Analysis of RNA-Seq expression data for endometrial stromal fibroblast primary cells (Kin *et al.*, 2016). (*A*) Percentage of differentially expressed genes in each species, separated by the age of the most recent common ancestor for each gene. The one-sided *p*-value calculated using Fisher's exact test is shown on the right, together with the number *N* of expressed genes in each species with an annotation in the NCBI HomoloGene database. (*B*) Enrichment of differentially expressed genes in each species, separated by the age of the most recent common ancestor. The enrichment was calculated by comparing the number of differentially expressed genes to the number of differentially expressed genes in an expression-matched background, with the $\log_2$(odds ratio) shown on the horizontal axis of the left panel and *p*-value calculated using Fisher's exact test for each comparison in the right panel. *N* is the total number of expressed genes in each species with an annotation in the NCBI HomoloGene database. (*C*) Gene Ontology analysis of differentially expressed genes. The *p*-value, calculated using Fisher's exact test, of overrepresentation or underrepresentation of differentially expressed genes in each Gene Ontology category compared to an expression-matched set of background genes is shown in red and blue, respectively.

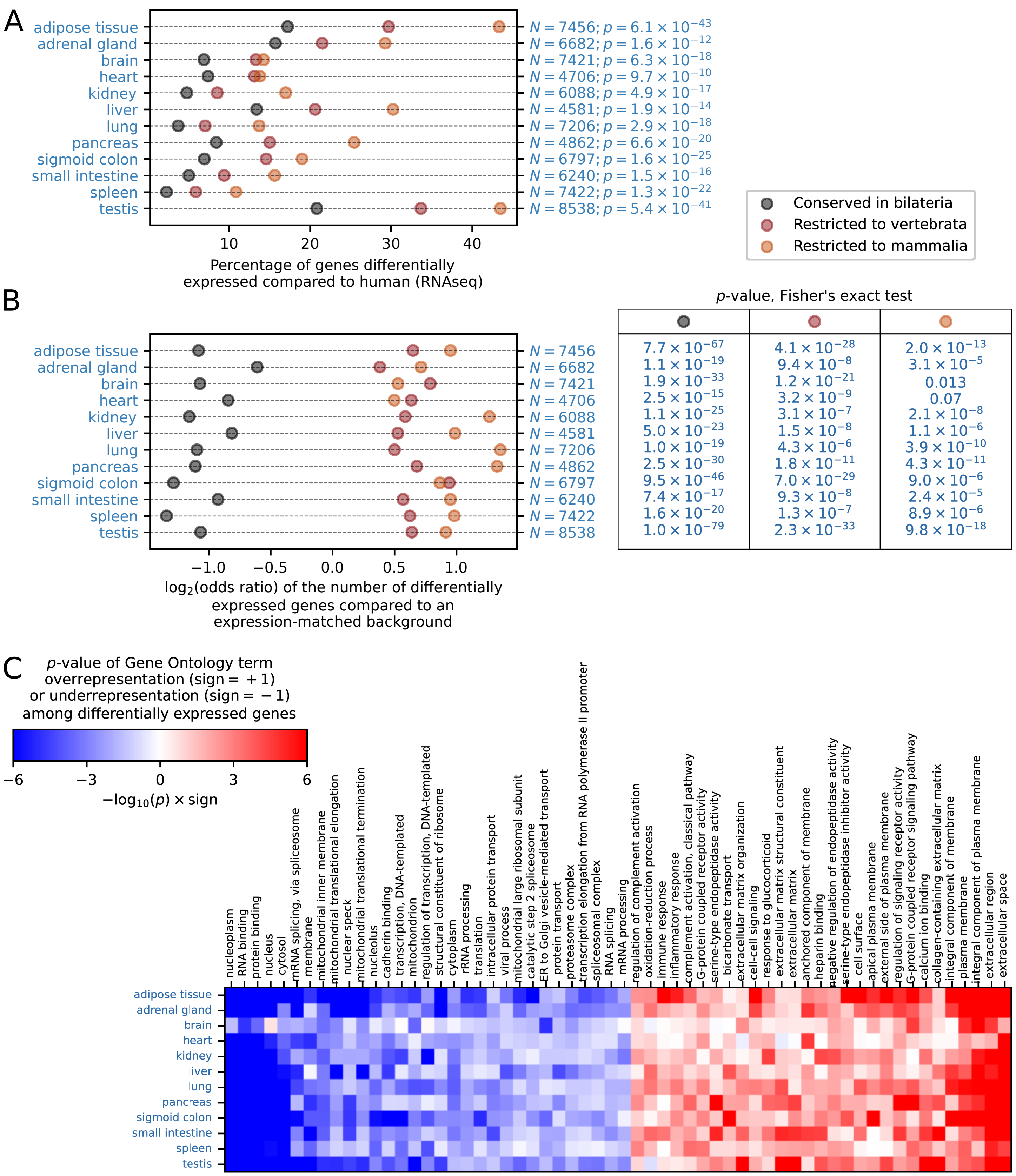
A
adipose tissue
adrenal gland
brain
heart
kidney
liver
lung
pancreas
sigmoid colon
small intestine
spleen
testis
N = 7456; p = 6.1 × 10^−43
N = 6682; p = 1.6 × 10^−12
N = 7421; p = 6.3 × 10^−18
N = 4706; p = 9.7 × 10^−10
N = 6088; p = 4.9 × 10^−17
N = 4581; p = 1.9 × 10^−14
N = 7206; p = 2.9 × 10^−18
N = 4862; p = 6.6 × 10^−20
N = 6797; p = 1.6 × 10^−25
N = 6240; p = 1.5 × 10^−16
N = 7422; p = 1.3 × 10^−22
N = 8538; p = 5.4 × 10^−41
10 20 30 40
Percentage of genes differentially expressed compared to human (RNAseq)
Conserved in bilateria
Restricted to vertebrata
Restricted to mammalia
B
N = 7456
N = 6682
N = 7421
N = 4706
N = 6088
N = 4581
N = 7206
N = 4862
N = 6797
N = 6240
N = 7422
N = 8538
−1.0 −0.5 0.0 0.5 1.0
log2(odds ratio) of the number of differentially expressed genes compared to an expression-matched background
p-value, Fisher's exact test
7.7 × 10^−67 | 4.1 × 10^−28 | 2.0 × 10^−13
1.1 × 10^−19 | 9.4 × 10^−8 | 3.1 × 10^−5
1.9 × 10^−33 | 1.2 × 10^−21 | 0.013
2.5 × 10^−15 | 3.2 × 10^−9 | 0.07
1.1 × 10^−25 | 3.1 × 10^−7 | 2.1 × 10^−8
5.0 × 10^−23 | 1.5 × 10^−8 | 1.1 × 10^−6
1.0 × 10^−19 | 4.3 × 10^−6 | 3.9 × 10^−10
2.5 × 10^−30 | 1.8 × 10^−11 | 4.3 × 10^−11
9.5 × 10^−46 | 7.0 × 10^−29 | 9.0 × 10^−6
7.4 × 10^−17 | 9.3 × 10^−8 | 2.4 × 10^−5
1.6 × 10^−20 | 1.3 × 10^−7 | 8.9 × 10^−6
1.0 × 10^−79 | 2.3 × 10^−33 | 9.8 × 10^−18
C
p-value of Gene Ontology term overrepresentation (sign = +1) or underrepresentation (sign = −1) among differentially expressed genes
−6 −3 0 3 6
−log10(p) × sign
nucleoplasm
RNA binding
protein binding
nucleus
cytosol
mRNA splicing, via spliceosome
membrane
mitochondrial inner membrane
mitochondrial translational elongation
nuclear speck
mitochondrial translational termination
nucleolus
cadherin binding
transcription, DNA-templated
mitochondrion
regulation of transcription, DNA-templated
structural constituent of ribosome
cytoplasm
rRNA processing
translation
intracellular protein transport
viral process
mitochondrial large ribosomal subunit
catalytic step 2 spliceosome
ER to Golgi vesicle-mediated transport
protein transport
proteasome complex
transcription elongation from RNA polymerase II promoter
spliceosomal complex
RNA splicing
mRNA processing
regulation of complement activation
oxidation-reduction process
immune response
inflammatory response
complement activation, classical pathway
G-protein coupled receptor activity
serine-type endopeptidase activity
bicarbonate transport
extracellular matrix organization
cell-cell signaling
response to glucocorticoid
extracellular matrix structural constituent
extracellular matrix
anchored component of membrane
heparin binding
negative regulation of endopeptidase activity
serine-type endopeptidase inhibitor activity
cell surface
apical plasma membrane
external side of plasma membrane
regulation of signaling receptor activity
G-protein coupled receptor signaling pathway
calcium ion binding
collagen-containing extracellular matrix
integral component of membrane
plasma membrane
integral component of plasma membrane
extracellular region
extracellular space

Supplemental Figure S13 (previous page).

Comparative analysis of RNA-Seq expression data for 12 matching tissues in human and mouse (The ENCODE Project Consortium, 2012). (*A*) Percentage of differentially expressed genes in each tissue, separated by the age of the most recent common ancestor for each gene. The one-sided *p*-value calculated using Fisher's exact test is shown on the right, together with the number *N* of expressed genes in each tissue with an annotation in the NCBI HomoloGene database. (*B*) Enrichment of differentially expressed genes in each tissue, separated by the age of the most recent common ancestor. The enrichment was calculated by comparing the number of differentially expressed genes to the number of differentially expressed genes in an expression-matched background, with the $\log_2$(odds ratio) shown on the horizontal axis of the left panel and *p*-value calculated using Fisher's exact test for each comparison in the right panel. *N* is the total number of expressed genes in each tissue with an annotation in the NCBI HomoloGene database. (*C*) Gene Ontology analysis of differentially expressed genes. The *p*-value, calculated using Fisher's exact test, of overrepresentation or underrepresentation of differentially expressed genes in each Gene Ontology category compared to an expression-matched set of background genes is shown in red and blue, respectively.

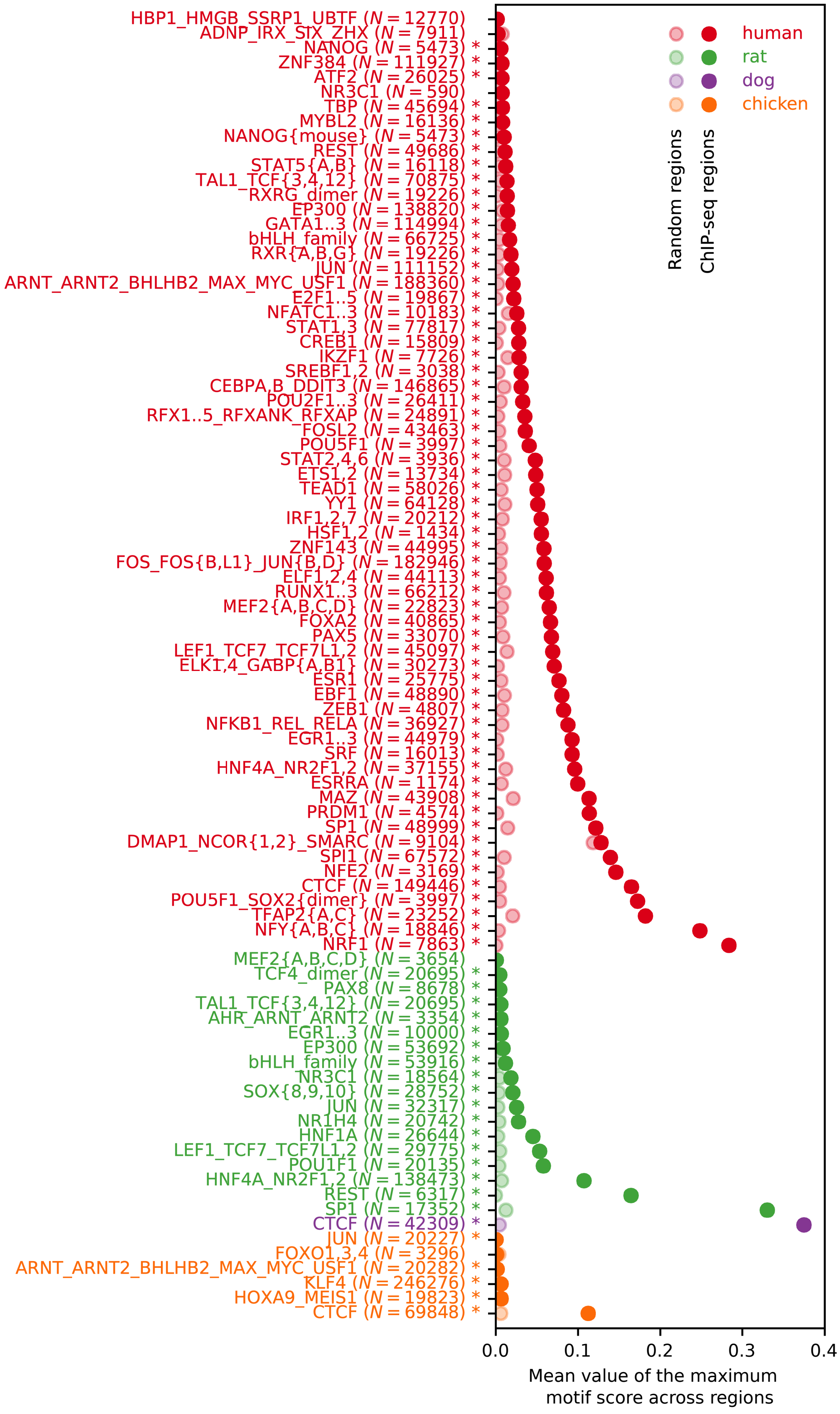

Supplemental Figure S14.

Evaluation of predicted transcription factor binding sites. The maximum TFBS score was calculated for each genomic region found in ChIP-seq experiments to be bound by transcription factors associated with the motif, and compared to an equal number of randomly selected genomic regions. The number *N* of ChIP-seq regions for each motif is shown. Motifs for which the maximum motif scores are significantly ($p <$ 0.05, Mann-Whitney *U* test) higher for ChIP-seq regions compared to background regions are indicated by an asterisk.

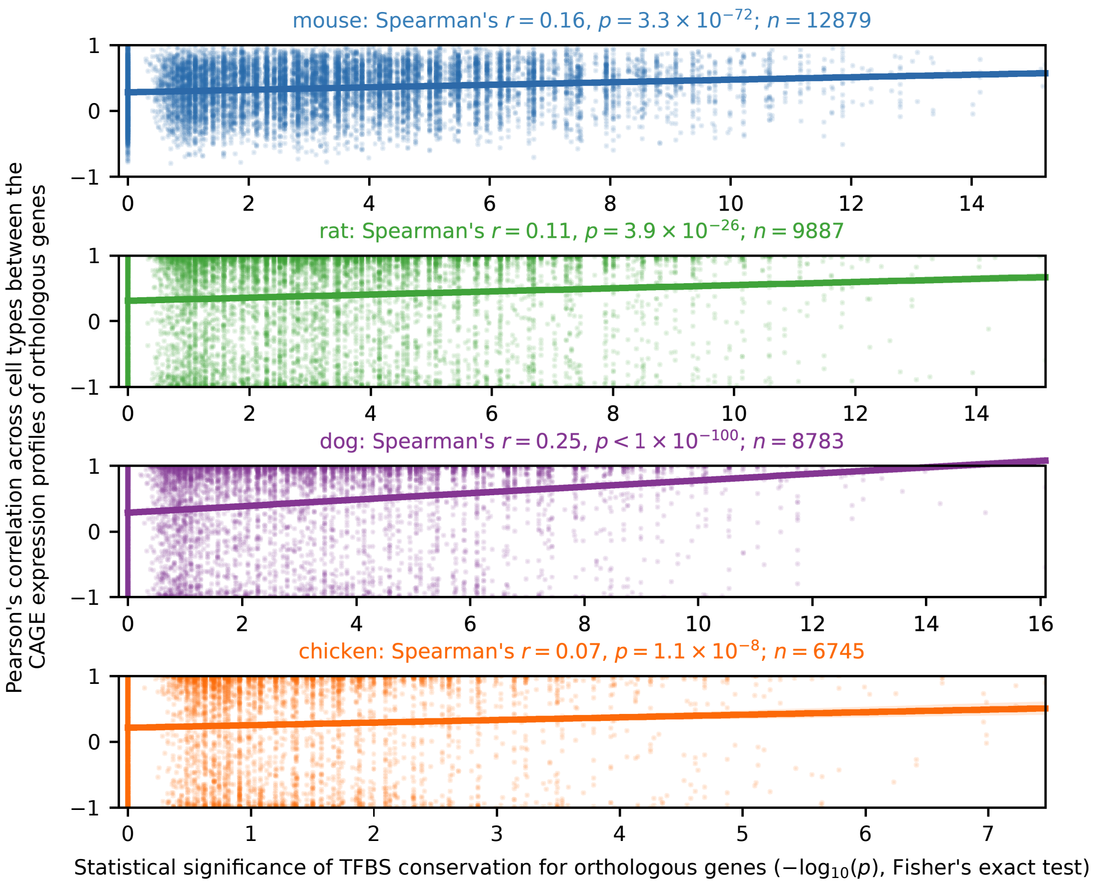

Supplemental Figure S15.

Pearson's correlation across cell types between orthologous genes, as a function of TFBS conservation in their promoter regions. TFBS conservation was evaluated as the *p*-value, calculated using Fisher's exact test, of co-occurence of binding sites, predicted by MotEvo, for the same transcription factors in the promoters of each orthologous gene pair.

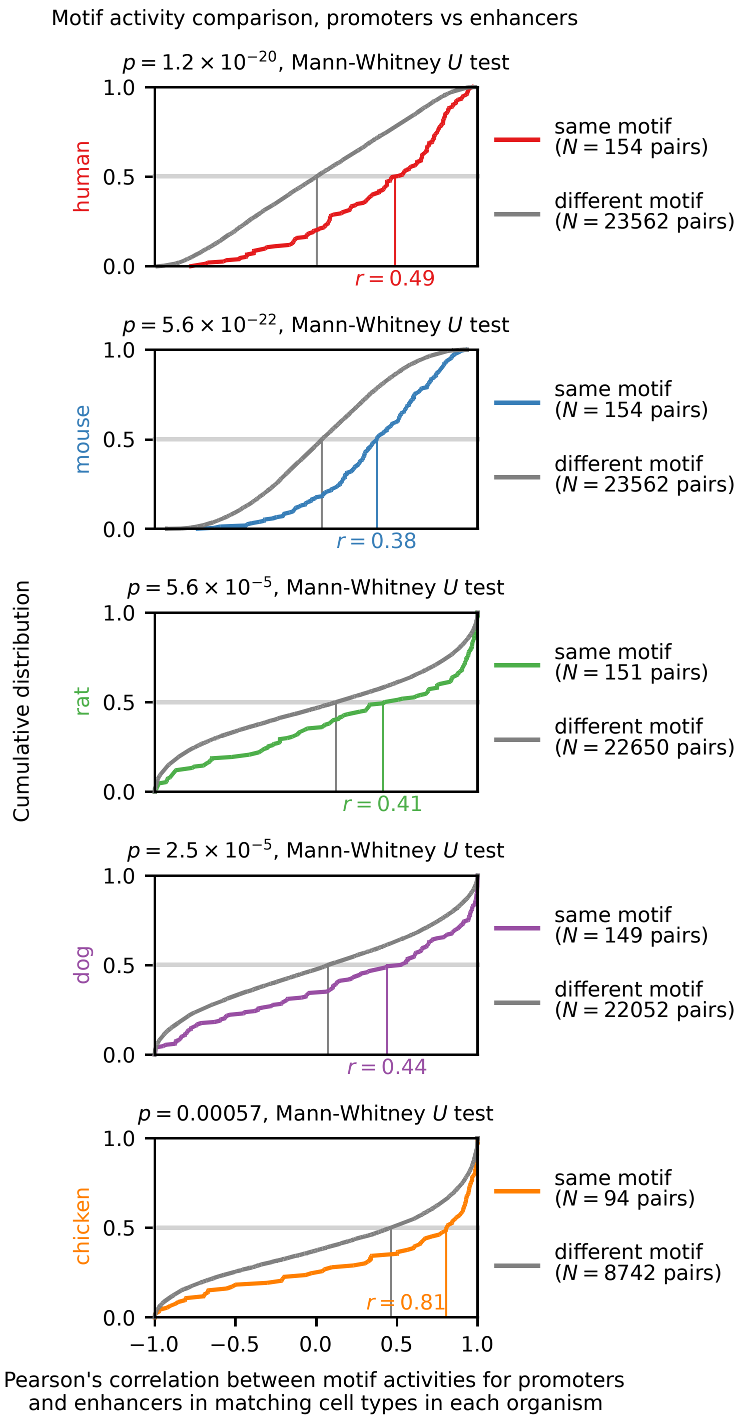

Supplemental Figure S16.

Cumulative distribution of the Pearson's correlation r across cell types in motif activity between promoters and enhancers in human, mouse, rat, dog, and chicken. The estimated median value of r is indicated on the horizontal axis of each graph. As a background distribution, we calculated the same correlation between pairs of different motifs in each of the five species. The Mann-Whitney U test p-value comparing the actual correlation values to the correlation values of the background distribution is shown for each species.

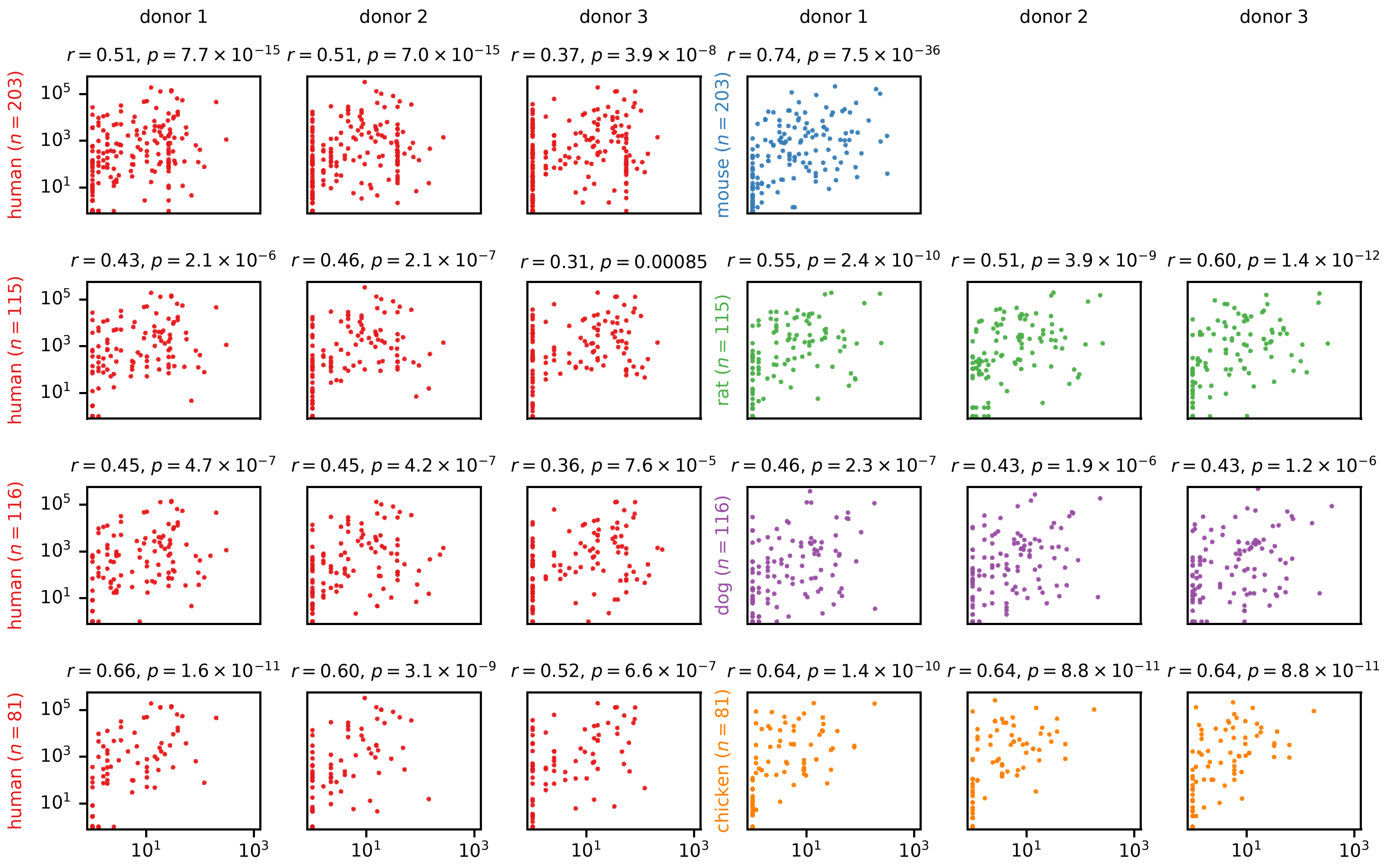

Supplemental Figure S17.

Comparison of CAGE expression levels of the pri-miRNA to the mature miRNA expression levels measured by sRNA sequencing in aortic smooth muscle cells in human, mouse, rat, dog, and chicken. To allow a direct comparison of the correlation values observed in human and each of the species, each row compares the CAGE and sRNA expression levels for the same set of miRNAs in human and mouse, rat, dog, or chicken.

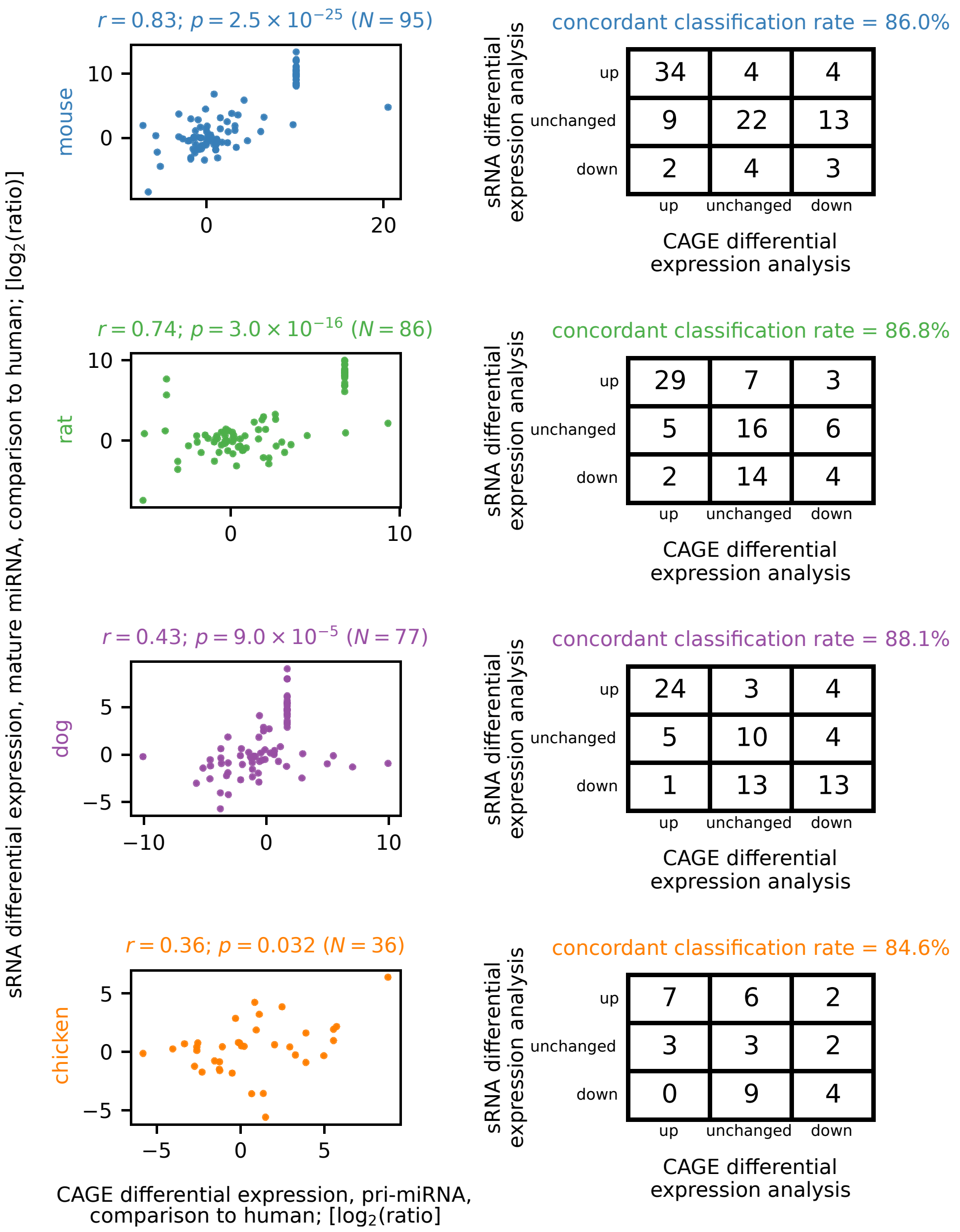

Supplemental Figure S18.

Comparison of differential expression analysis of pri-miRNAs and mature miRNAs. (*left*) Comparison of log_2 expression ratios obtained in differential expression analysis performed on the expression levels of pri-miRNAs (using CAGE sequencing data) and mature miRNA (using sRNA sequencing data). (*right*) Number of differentially expressed miRNAs found in differential expression analysis of pri-miRNA (CAGE) and mature miRNA (sRNA) expression data. The concordant classification rate is calculated as the number of miRNAs that are significantly up- or down-regulated compared to human both in CAGE differential expression analysis and in sRNA differential expression analysis, divided by the total number of miRNAs differentially expressed in both CAGE and sRNA differential expression analysis.

.
