# Supplementary material for: Comparative transcriptomics of primary cells in vertebrates
Source: Genome Res. 2020 Jul;30(7):951–61. doi: 10.1101/gr.255679.119 (PMC7397866; doi:10.1101/gr.255679.119)
Supplement: Supplemental Material [file supp_gr.255679.119_Supplemental_Methods.docx]

# Supplemental Methods

## Gene expression quantitation

For each promoter region for each gene, we found the total CAGE tag count, summing over all libraries included in this study. For each gene, the region with the highest total CAGE tag count was identified as the dominant promoter, and the position with the highest CAGE tag count within this region as the representative transcription start site of the gene. For each gene, the CAGE expression level in a CAGE library was defined as the sum of CAGE tags across all regions associated with the gene. CAGE expression data were normalized to tags-per-million (t.p.m.) by dividing by the total sum of CAGE tags associated with genes, and multiplying by 1,000,000. Genes with an expression level of at least 10 t.p.m. were included in the analysis.

## RNA-seq expression data

ENCODE (The ENCODE Project Consortium 2012) RNA-seq gene expression data were downloaded from the ENCODE portal (Davis et al. 2018) (https://www.encodeproject.org/) as gene quantifications in tsv format with sample annotations in json format for the following accession numbers: ENCFF005MLW, ENCFF051UVH, ENCFF084WWG, ENCFF115BZB, ENCFF122LQH, ENCFF134RPA, ENCFF143IYG, ENCFF152DMV, ENCFF162KBI, ENCFF201BIE, ENCFF203RCU, ENCFF227RBV, ENCFF280HQA, ENCFF362GHJ, ENCFF375LAX, ENCFF421SLK, ENCFF428HNN, ENCFF442XPO, ENCFF456GZP, ENCFF485EYP, ENCFF507RNA, ENCFF508QDF, ENCFF509LQW, ENCFF517ZTC, ENCFF547TAC, ENCFF549OGG, ENCFF603OKL, ENCFF625HJC, ENCFF637YVK, ENCFF664JKG, ENCFF677ZIY, ENCFF686JQP, ENCFF750HMK, ENCFF780DAF, ENCFF784ZTQ, ENCFF798PSE, ENCFF804BIT, ENCFF804WTK, ENCFF809RAX, ENCFF850LMK, ENCFF866LBS, ENCFF878LJT, ENCFF878UHQ, ENCFF908GIP, ENCFF911YUO, ENCFF916CFV, ENCFF926FDN, ENCFF937HOV. RNA-seq gene expression count tables for endometrial stromal fibroblast (Kin et al. 2016), their normalized expression levels in tags per million, and gene ortholog associations were downloaded from the NCBI Gene Expression Omnibus under accession number GSE67659.

## Pairwise genome alignments

Preprocessing of the genome sequences and postprocessing of the alignments was performed using partitionSequence.pl, blastz-normalizeLav, lavToPsl, axtChain, chainAntiRepeat, chainMergeSort, chainPreNet, chainNet, netSyntenic, netFilter, netToAxt, axtSort, and axtToMaf, which are part of the UCSC Genome Browser bioinformatics utilities (Kuhn et al. 2013) release 366 (June 5, 2018). First, for each pairwise alignment we used partitionSequence.pl to split the target and query genome sequences into segments of 20 Mb, with the target sequence segments overlapping by 10 kb. Sequences were aligned using lastz (Harris 2007) version 1.03.66 with parameters shown in Supplemental Table S15. Alignment coordinates were corrected using blastz-normalizeLav and converted to .psl format using lavToPsl. Alignments were chained using axtChain with parameters shown in Supplemental Table S15 and further processed using chainAntiRepeat, chainMergeSort, chainPreNet, chainNet, netSyntenic, and netFilter. The best alignments were extracted using netToAxt, followed by axtSort and axtToMaf to generate a .maf (multiple alignment format) file.

## Genome-wide TFBS prediction

For human and mouse, alignments for human, macaque, mouse, rat, dog, horse, cow, opossum, and chicken were extracted from the 100-way (human) and 30-way (mouse, rat, dog, and chicken) multiple genome alignments; for rat, dog, and chicken, we used the 30-way multiple genome alignments described in the main Methods section of this manuscript. Extracted alignments were improved as described previously (Arner et al. 2015) using the T-Coffee [(Notredame et al. 2000)](http://f1000.com/work/citation?ids=23986&pre=&suf=&sa=0) multiple sequence aligner version 9.01. We ran MotEvo [(Arnold et al. 2012)](http://f1000.com/work/citation?ids=462788&pre=&suf=&sa=0) version 1.01 on these alignments as described previously (Arner et al. 2015), but using the 190 motifs in the SwissRegulon [(Pachkov et al. 2013)](http://f1000.com/work/citation?ids=3419764&pre=&suf=&sa=0) release of July 13, 2015, and with a background probability for the four nucleotides that was calculated by counting their frequency across the genome.

## Motif activity analysis

The scripts make_profile.py, associate_tfbs.py, and calculate_motif_activity.py, available at <http://fantom.gsc.riken.jp/5/suppl/Alam_et_al_2020/> and in the Supplemental Code, provide an implementation of the motif activity analysis methodology described previously (Suzuki et al. 2009). The density of predicted transcription factor binding sites with respect to the representative transcription start site was calculated for each motif using the script make_profile.py with default options. Using the calculated density, predicted binding sites were associated with the dominant promoter of each gene by the script associate_tfbs.py with default options. The density of predicted transcription factor binding sites in a ±500 base pair window around enhancers was calculated using the script make_profile.py with options --upstream=500 --downstream=500 --symmetric. Using the calculated density, predicted binding sites were associated with each enhancer by the script associate_tfbs.py with default options.

We then selected motifs with at least 50 predicted binding sites both for promoters and in enhancers in each pair of species, resulting in 154 (human-mouse), 151 (human-rat), 149 (human-dog), and 94 (human-chicken) motifs. Motif activities were calculated for promoters and enhancers separately for the selected motifs using the script calculate_motif_activity.py with option ‘-n 0’.

## MicroRNA analysis

We used release 21 of the miRBase database [(Kozomara and Griffiths-Jones 2014)](http://f1000.com/work/citation?ids=316451&pre=&suf=&sa=0), lifted over for rat and chicken to genome assembly rn6 and galGal5 respectively, as our reference set of known miRNAs; one pre-miRNA in rat and 26 pre-miRNAs in chicken could not be lifted over and were dropped. Pre-miRNAs were classified as robust or permissive (Supplemental Table S9) as described previously [(De Rie et al. 2017)](http://f1000.com/work/citation?ids=4081215&pre=&suf=&sa=0).

Candidate novel miRNAs were identified using miRDeep2 [(Friedländer et al. 2012)](http://f1000.com/work/citation?ids=463493&pre=&suf=&sa=0), resulting in 229 (rat), 249 (dog), and 180 (chicken) predicted pre-miRNAs, including 169 (rat), 179 (dog), and 128 (chicken) known pre-miRNAs and 59 (rat), 74 (dog), and 55 (chicken) candidate novel pre-miRNAs (Supplemental Table S10).

Promoters for miRNAs in rat, dog, and chicken were identified using the same approach as applied previously for human and mouse [(De Rie et al. 2017)](http://f1000.com/work/citation?ids=4081215&pre=&suf=&sa=0), using transcripts annotated for rat, dog, or chicken in the NCBI Entrez Gene database [(Brown et al. 2015)](http://f1000.com/work/citation?ids=111772&pre=&suf=&sa=0), downloaded on 13 November 2017, as candidate primary miRNAs, and the permissive set of CAGE transcription initiation peaks (Kawaji 2020) as candidate promoters. Identified promoters for all robust miRNAs in mouse, rat, dog, and chicken were curated manually by two annotators (Supplemental Table S12).

## Supplemental References

Davis CA, Hitz BC, Sloan CA, Chan ET, Davidson JM, Gabdank I, Hilton JA, Jain K, Baymuradov UK, Narayanan AK, et al. 2018. The Encyclopedia of DNA elements (ENCODE): data portal update. *Nucleic Acids Res* **46**(D1): D794–D801.

Friedländer MR, Mackowiak SD, Li N, Chen W, Rajewsky N. 2012. miRDeep2 accurately identifies known and hundreds of novel microRNA genes in seven animal clades. *Nucleic Acids Res* **40**: 37–52.

Harris RS. 2007. Improved pairwise alignment of genomic DNA. Ph.D. thesis. Pennsylvania State University, State College, PA.

Kawaji H. 2020. Transcription initiation peaks based on FANTOM5 CAGE data on rn6, canFam3, and galGal5. Zenodo doi: 10.5281/zenodo.3856413.

Notredame C, Higgins DG, Heringa J. 2000. T-Coffee: A novel method for fast and accurate multiple sequence alignment. *J Mol Biol* **302**: 205–217.
